# CUL4-DDB1-CRBN E3 Ubiquitin Ligase Regulates Proteostasis of ClC-2 Chloride Channels: Implication for Aldosteronism and Leukodystrophy

**DOI:** 10.3390/cells9061332

**Published:** 2020-05-26

**Authors:** Ssu-Ju Fu, Meng-Chun Hu, Yi-Jheng Peng, Hsin-Yu Fang, Cheng-Tsung Hsiao, Tsung-Yu Chen, Chung-Jiuan Jeng, Chih-Yung Tang

**Affiliations:** 1 Department of Physiology, College of Medicine, National Taiwan University, Taipei 10051, Taiwan; d01441001@ntu.edu.tw (S.-J.F.); mengchun@ntu.edu.tw (M.-C.H.); yijhengp@usc.edu (Y.-J.P.); hyfang2@illinois.edu (H.-Y.F.); hsiaoct26@gmail.com (C.-T.H.); 2 Department of Neurology, Taipei Veterans General Hospital, Taipei 12217, Taiwan; 3 Center for Neuroscience and Department of Neurology, University of California, Davis, CA 95616, USA; tycchen@ucdavis.edu; 4 Institute of Anatomy and Cell Biology, School of Medicine, National Yang-Ming University, Taipei 12212, Taiwan; 5 Brain Research Center, National Yang-Ming University, Taipei 12212, Taiwan; 6 Graduate Institute of Brain and Mind Sciences, College of Medicine, National Taiwan University, Taipei 10051, Taiwan

**Keywords:** channelopathy, cullin E3 ubiquitin ligase, polyubiquitination, proteasomal degradation, MG132, MLN4924, lenalidomide

## Abstract

Voltage-gated ClC-2 channels are essential for chloride homeostasis. Complete knockout of mouse ClC-2 leads to testicular degeneration and neuronal myelin vacuolation. Gain-of-function and loss-of-function mutations in the ClC-2-encoding human *CLCN2* gene are linked to the genetic diseases aldosteronism and leukodystrophy, respectively. The protein homeostasis (proteostasis) mechanism of ClC-2 is currently unclear. Here, we aimed to identify the molecular mechanism of endoplasmic reticulum-associated degradation of ClC-2, and to explore the pathophysiological significance of disease-associated anomalous ClC-2 proteostasis. In both heterologous expression system and native neuronal and testicular cells, ClC-2 is subject to significant regulation by cullin-RING E3 ligase-mediated polyubiquitination and proteasomal degradation. The cullin 4 (CUL4)-damage-specific DNA binding protein 1 (DDB1)-cereblon (CRBN) E3 ubiquitin ligase co-exists in the same complex with and promotes the degradation of ClC-2 channels. The CRBN-targeting immunomodulatory drug lenalidomide and the cullin E3 ligase inhibitor MLN4924 promotes and attenuates, respectively, proteasomal degradation of ClC-2. Analyses of disease-related ClC-2 mutants reveal that aldosteronism and leukodystrophy are associated with opposite alterations in ClC-2 proteostasis. Modifying CUL4 E3 ligase activity with lenalidomide and MLN4924 ameliorates disease-associated ClC-2 proteostasis abnormality. Our results highlight the significant role and therapeutic potential of CUL4 E3 ubiquitin ligase in regulating ClC-2 proteostasis.

## 1. Introduction

Voltage-gated ClC-2 chloride (Cl^−^) channels are broadly expressed in virtually all tissues, with particularly abundant expression levels in neurons, glial cells, and epithelia cells [1,2,3,4,5]. The primary physiological activation mechanism of the ClC-2 channel involves membrane hyperpolarization, and osmotic cell swelling may also activate this voltage-dependent Cl^−^ channel gating process [6,7]. Moreover, complete knockout of ClC-2 in mice leads to severe retinal and testicular degeneration [8,9], as well as prominent fluid accumulation and myelin vacuolation in central neurons [10]. Together, these observations suggest that ClC-2 channels may regulate ion homeostasis in narrow extracellular spaces, such as those in blood–retinal, blood–testis, and blood–brain barriers.

Diseases caused by dysfunction of ion channels are collectively known as channelopathy. Various mutations in the human *CLCN2* gene, which encodes the ClC-2 channel, have been associated with distinct types of genetic diseases. In primary aldosteronism, gain-of-function mutations in the *CLCN2* gene lead to enhanced Cl^−^ efflux and therefore membrane depolarization in aldosterone-producing adrenal glomerulosa cells, manifesting as constitutive aldosterone secretion, hypertension, and hypokalemia [11,12,13,14,15]. On the other hand, loss-of-function mutations in the *CLCN2* gene have been linked to a type of leukodystrophy (white matter disorder), *CLCN2*-related leukoencephalopathy, characterized by intramyelinic edema in the brain [16,17,18], which is reminiscent of the myelin vacuolation found in ClC-2 knockout mice [10]. Moreover, another type of leukodystrophy, megalencephalic leukoencephalopathy with subcortical cysts, is associated with genetic mutations in the ClC-2-binding proteins GlialCAM and megalencephalic leukoencephalopathy with subcortical cysts 1 (MLC1), whose disease-related defects significantly disrupt the subcellular localization of ClC-2 channels in astrocytes and oligodendrocytes, as well as reducing ClC-2 protein stability at the plasma membrane [19,20,21]. Interestingly, consistent with the presence of testicular degeneration in *Clcn2*-deficient mice [9], infertility was observed in a leukodystrophy patient carrying a loss-of-function mutation in the *CLCN2* gene [22]. 

Biophysical analyses indicate that functional expression of Cl^−^ currents is notably enhanced and diminished in aldosteronism- and leukodystrophy-associated ClC-2 mutant channels, respectively. The mechanism underlying the enhanced cell surface Cl^−^ conductance in aldosteronism can be attributed to altered voltage-dependent gating properties that increase the current amplitude of mutant ClC-2 channels [12,13,14]. In contrast, leukodystrophy-associated mutations result in altered voltage-dependent gating properties that reduce the current amplitude of mutant ClC-2 channels [18]. Importantly, leukodystrophy-associated mutations also lead to reduced ClC-2 protein levels that may involve defective protein stability and impaired membrane trafficking [16,18]. It remains unclear whether aldosteronism-causing mutations may also affect the biochemical property of ClC-2 channels by, for example, promoting ClC-2 protein expression. 

The regulation of protein homeostasis (proteostasis) entails both translational and post-translational mechanisms governing protein conformation, stability, and subcellular localization [23,24]. For membrane proteins, such as ClC-2, one of the key proteostasis mechanisms is mediated by the endoplasmic reticulum (ER) quality control system, which works in a stringent way to selectively remove misfolded proteins via proteasomal degradation, a process known as ER-associated degradation [25,26]. The molecular basis of the protein degradation process for ClC-2 protein is virtually unknown. In ER-associated degradation, misfolded proteins are subject to a concerted activity of the ubiquitination machinery that includes the ubiquitin activating enzyme (E1), the ubiquitin conjugating enzyme (E2), and the ubiquitin ligase (E3) [26,27,28]. In order to elucidate the protein degradation mechanism of ClC-2 channels, in this study, we aimed to identify the molecular nature of the E3 ubiquitin ligase of ClC-2 channels and to explore the pathophysiological role of proteasomal degradation in the abovementioned ClC-2 channelopathies. 

## 2. Materials and Methods

### 2.1. cDNA Constructs

Mouse ClC-2 cDNA was subcloned into the pcDNA3-Flag vector (Invitrogen, Carlsbad, CA, USA) to generate the N-terminal Flag-tagged ClC-2 construct. Myc-tagged ClC-2 in the pcDNA3 vector was generated by inserting the epitope sequence between the residues V420 and E421 in the extracellular linker between helices L and M. Other cDNA constructs employed in this study include pcDNA3.1-Flag dominant-negative human cullin 1/2/3/4A/4B/5 (Addgene 15,818–15,823, Watertown, MA, USA), pcDNA3-Myc human cullin 4A/4B (Addgene 19,951, 19,922, Watertown, MA, USA), pcDNA3-HA lysine-less human ubiquitin (kindly provided by Dr. Chihiro Sasakawa, University of Tokyo, Tokyo, Japan), pcDNA3-Flag human DDB1 (Addgene 19,918, Watertown, MA, USA), pcDNA3-Flag human DDB2 (kindly provided by Dr. Show-Li Chen, National Taiwan University, Taipei, Taiwan), and pcDNA3-HA rat cereblon (kindly provided by Dr. Chul-Seung Park, Gwangju Institute of Science and Technology, Gwangju, Korea).

### 2.2. Preparation of Animal Samples

Wistar rats and C57BL/6 mice were handled in accordance with the National Institute of Health Guide for the Care and Use of Laboratory Animals (NIH Publications No. 80-23, revised 1996, Bethesda, MD, USA). All procedures involving animals were performed in conformity with the animal protocol approved by the Institutional Animal Care and Use Committee (IACUC), College of Medicine, National Taiwan University.

For the preparation of brain homogenates, rat brain tissues were homogenized with a motor-driven glass-Teflon homogenizer in ice-cold dissociation buffer ((in mm) 320 sucrose, 1 MgCl_2,_ 0.5 CaCl_2,_ 1 NaHCO_3,_ 1 phenylmethylsulfonyl fluoride (PMSF) and 1 mg/L leupeptin) and the cell debris was removed by centrifugation at 1400× *g* for 10 min. The supernatant was saved, and the pellet was resuspended by homogenization in ice-cold dissociation buffer and pelleted again. The remaining pellet was discarded, and the combined supernatants were pelleted (13,800× *g* for 10 min) again. The final pellet was resuspended in buffer A ((in mm) 100 NaCl, 4 KCl, 2.5 EDTA, 20 NaHCO_3_, 20 Tris-HCl, pH 7.5, plus 1 PMSF, 1 Na_3_VO_4_, 1 NaF, 1 β-glycerophosphate) containing 1% Triton X-100 and the complete protease inhibitor cocktail (Roche Applied Science, Penzberg, Germany).

Dissociated cortical neurons were prepared from the embryos of 18-day-old pregnant rats using a previously described protocol [29] with a minor modification [30]. In brief, cerebral cortices were dissected from the forebrains of embryonic day 18 (E18) pups, whose brains were removed and placed in Hank’s balanced salt solution containing 10 mm HEPES (pH 7.4) and 1 mm sodium pyruvate, followed by dissociation with 0.25% trypsin solution. The dissociated cells were plated on 6-cm culture dishes coated with poly-D-lysine (1 mg/mL) (Sigma, St. Louis, MO, USA) and laminin (15 g/mL) (Sigma, St. Louis, MO, USA). Cultures were maintained in the Neurobasal media supplemented with B27 (2%) and glutamax I (0.5 mm) (Invitrogen, Carlsbad, CA, USA) in a humidified 5% CO_2_ incubator at 37 °C. Ten days in vitro (DIV10) cortical cultures were employed for treatment with MLN4924. 

Testes dissected from mice were homogenized in tissue lysis buffer ((in mm) (in mm) 20 Tris-HCl, pH 7.9, 137 NaCl, 10 NaF, 5 EDTA, 1 Na_3_VO_4_, 1 Na_4_P_2_O_7_, 0.1 β-glycerophosphate, 10% glycerol, 1% Triton X-100, 5 dithiothreitol (DTT), 1 PMSF, 10 µg/mL Leupeptin). The homogenates were then incubated on ice for 1 h, followed by centrifugation at 13,000× *g* for 30 min at 4 °C to collect testis lysates.

For the preparation of the primary culture of mouse Leydig cells, testes collected from four- to five-week-old mice were decapsulated and incubated with 0.5 mg/mL collagenase type IV (Sigma, St. Louis, MO, USA) in Dulbecco’s modified Eagle’s medium (DMEM)/F12 at room temperature for 15 min with gentle shaking, followed by filtering through 70-μm meshes (Falcon) to separate seminiferous tubules, and centrifugation at 800× *g* for 2 min to collect interstitial cells. Leydig cells were resuspended and cultured in DMEM/F12 containing 10% fetal bovine serum (FBS) and antibiotics, followed by incubation at 37 °C for 72 h.

### 2.3. Cell Culture and DNA Transfection

Human embryonic kidney (HEK) 293T cells were grown in DMEM supplemented with 2 mm glutamine, 10% heat-inactivated fetal bovine serum (Hyclone), 100 units/mL penicillin, and 50 μg/mL streptomycin, and were maintained at 37 °C in a humidified incubator with 95% air and 5% CO_2_. Mouse MA-10 Leydig cells were maintained in DMEM/F12 supplemented with 10% fetal bovine serum and 20 mm HEPES. Transient transfection was performed by using the Lipofectamine 2000 reagent (Invitrogen, Carlsbad, CA, USA). Cells were plated onto 12-well plates 24 h before transfection. Various expression constructs were incubated with the transfection reagent for 20 min at room temperature, and DNA-lipofectamine diluted in Opti-MEM (Invitrogen, Carlsbad, CA, USA) was added to culture wells. After 6 h of incubation at 37 °C, the medium was changed and the culture cells were maintained in the 37 °C incubator for 48 h. Where indicated, cycloheximide (Sigma, St. Louis, MO, USA), MG132 (Sigma, St. Louis, MO, USA), MLN4924 (kindly provided by Dr. Kuo-How Huang, National Taiwan University Hospital, Taipei, Taiwan), or lenalidomide (Sigma, St. Louis, MO, USA) were applied to the culture medium.

### 2.4. RNA Interference

Lentivirus-based shRNA constructs (subcloned into the pLKO vector) targeting specific mouse or human ClC-2 (5′-CCTAGCTCCGAGACATCTATC-3′; 5′-GTCACAGCACAGGGTGTTAAA-3′), CUL4A (5′-GCAGAACTGATCGCAAAGCAT-3′), CUL4B (5′-GCCATGAAAGAAGCATTTGAA-3′), CRBN (5′-CCTACCAAGTTCAAGAGCATA-3′; 5′-CCAGAAACATCTACTTGGGTA-3′), or DDB1 (5′-GCCTGCATCCTGGAGTATAAA-3′) sequences, as well as the control shRNA for LacZ (5′-TGTTCGCATTATCCGAACCAT-3′), were purchased from National RNAi Core Facility, Taiwan. Recombinant lentivirus was generated by co-transfecting HEK293T cells with the packaging plasmid pCMV-ÄR8.91, the envelope plasmid pMD.G, and shRNA-expressing constructs via the jetPRIME transfection reagent (Polyplus-transfection). Then, 48 h after transfection, the medium was collected on a daily basis (stored at −80 °C; followed by the application of new medium to HEK293T cells) for three consecutive days. The collected media containing lentiviral particles were centrifuged at 3000× *g* for 5 min, and supernatants were harvested and filtered (0.22 μm). MA-10 cells were maintained in the freshly collected viral supernatants in the presence of 8 μg/mL polybrene (Sigma, St. Louis, MO, USA) for at least 48 h, followed by incubation with a selection medium containing puromycin (5 µg/mL) for at least 48 h. 

### 2.5. Immunoblotting

Cells were washed twice with ice-cold Dulbecco’s phosphate-buffered saline (D-PBS) ((in mm) 137 NaCl, 2.7 KCl, 4.3 Na_2_HPO_4_ 2H_2_O, 1.4 KH_2_PO_4_, pH 7.3) supplemented with 2 mm EDTA, and resuspended in a lysis buffer ((in mm) 150 NaCl, 5 EDTA, 50 Tris-HCl pH 7.6, 1% Triton X-100) containing the complete protease inhibitor cocktail. After adding the Laemmli sample buffer to the lysates, samples were sonicated on ice (three times for five seconds each) and heated at 70 °C for 5 min. Samples were then separated by 7.5–10% SDS-PAGE, electrophoretically transferred to nitrocellulose membranes, and detected using mouse anti-β-actin (1:5000; Millipore, Burlington, MA, USA), rabbit-anti-ClC-2 (1:1000; Alomone, Jerusalem, Israel), rabbit anti-CUL4A (1:2000; GeneTex, Irvine, CA, USA), rabbit anti-CUL4B (1:1000; Proteintech, Rosemont, IL, USA), rabbit anti-CRBN (1:1000; Aviva Systems Biology, San Diego, CA, USA), rabbit anti-DDB1 (1:3000; GeneTex, Irvine, CA, USA), rabbit anti-Flag (1:5000; Sigma, St. Louis, MO, USA), rabbit anti-glyceraldehyde-3-phosphate dehydrogenase (GAPDH) (1:5000; GeneTex, Irvine, CA, USA), rat anti-HA (1:5000; Roche, Basel, Switzerland), mouse anti-Myc (1:5000; clone 9E10), or rabbit anti-α-tubulin (1:5000; GeneTex, Irvine, CA, USA) antibodies. Blots were then exposed to horseradish peroxidase-conjugated anti-mouse/rabbit IgG (1:5000; Jackson ImmunoResearch, West Grove, PA, USA), and revealed by an enhanced chemiluminescence detection system (Thermo Scientific, Waltham, MA, USA). Acquisition of chemiluminescent signals from immunoblots was achieved by using the UVP AutoChemi image system (Ultra-Violet Products, Upland, CA, USA). Results shown are representative of at least three independent experiments. Uncropped images of immunoblots are shown in Figure S1.

For quantitative analyses, data were collected from at least three independent experiments performed in duplicates or triplicates. Densitometric scans of immunoblots were quantified by using ImageJ (National Institute of Health, Bethesda, MD, USA). For a given immunoblot containing multiple lanes of protein signals associated with the same experimental condition addressing a specific issue, protein density was first standardized as the ratio of the densitometric signal of the protein of interest to that of the cognate loading control. Standardized protein density values of all the control groups were then used for calculating the mean control protein density. Standardized protein density values of individual protein lanes, treatment and control groups alike, were subsequently normalized with respect to the mean control protein density. For a given experimental condition with multiple repeats, normalized protein density values from multiple immunoblots were pooled together for statistical analyses. 

### 2.6. Co-Immunoprecipitation 

Cells were incubated at 37 °C in the presence of 10 μM MG132 for 24 h. Cells were solubilized in ice-cold immunoprecipitation (IP) buffer ((in mm) 100 NaCl, 4 KCl, 2.5 EDTA, 20 NaHCO_3_, 20 Tris-HCl, pH 7.5, 1 DTT, 1 PMSF, 1% Triton X-100) containing the protease inhibitor cocktail. Insolubilized materials were removed by centrifugation. Solubilized lysates were pre-cleared with protein G sepharose beads (GE Healthcare Biosciences, Piscataway Township, NJ, USA) for 1 h at 4 °C, and then incubated for 16 h at 4 °C with protein G sepharose beads pre-coated with the anti-Myc or anti-HA antibody. Beads were gently spun down and washed twice in a wash buffer ((in mm) 100 NaCl, 4 KCl, 2.5 EDTA, 20 NaHCO_3_, 20 Tris-HCl, pH 7.5) supplemented with 0.1% Triton X-100, and then twice with the wash buffer. The immune complexes were eluted from the beads by heating at 70 °C for 5 min in the Laemmli sample buffer. 

### 2.7. Cycloheximide Chase

At 24 h post-transfection, cells were subject to treatment with cycloheximide (100 μg/mL) for 0–8 h, followed by immunoblotting. Quantitative analyses of the ClC-2 protein degradation time course were implemented by standardizing protein densities as the ratio to the cognate loading control, followed by the normalization of various cycloheximide-treated groups with respect to the corresponding control group at 0 h. 

### 2.8. Protein Ubiquitination Analyses

Cells were incubated at 37 °C in the presence of 10 μM MG132 for 24 h. Cells were then solubilized in the IP buffer supplemented with 2.5 mg/mL N-ethylmaleimide to inactivate deubiquitinating enzymes, followed by immunoprecipitation. 

### 2.9. Immunofluorescence

Cells grown on coverslips were rinsed in ice-cold phosphate buffered saline (PBS, Arlington County, VA, USA) and fixed for 20 min with 4% paraformaldehyde in PBS at room temperature. After being washed with cold PBS, fixed cells were permeabilized and blocked with a blocking buffer (5% normal goat serum in 20 mm phosphate buffer, pH 7.4, 0.1% (v/v) Triton X-100, and 0.45 M NaCl) for 60 min at 4 °C. Cells were then immunolabeled overnight at 4 °C with a 1:200 dilution (in the blocking buffer) of rabbit anti-ClC-2 and mouse anti-CRBN (Proteintech, Rosemont, IL, USA) antibodies. Alexa Fluor 488-conjugated anti-rabbit IgG and Alexa Fluor 568-conjugated anti-mouse IgG (1:200; Molecular Probes, Eugene, OR, USA) were used as secondary antibodies. Nuclei were stained with DAPI. After the final wash, coverslips were mounted in a mounting medium (4% n-propylgallate, 90% glycerol, 0.1 M carbonate buffer, pH 9.2). Fluorescence images were viewed and acquired with a confocal microscope (TCS SP8, Leica, Wetzlar, Germany).

### 2.10. Cell Surface Biotinylation

Cells were rinsed with ice-cold D-PBS supplemented with 0.5 mm CaCl_2_, 2 mm MgCl_2_, incubated in 1 mg/mL sulfo-NHS-LC-biotin (Thermo Scientific, Waltham, MA, USA) in D-PBS at 4 °C for 1 h with gentle rocking on an orbital shaker, and subject to a quenching procedure by removing the biotin reagents and rinsing 3 times with 100 mm glycine in PBS, followed by once in Tris buffered saline ((in mm) 20 Tris-HCl, 150 NaCl, pH 7.4). Cells were solubilized in ice-cold lysis buffer ((in mm) 150 NaCl, 50 Tris-HCl, 1% Triton X-100, 5 EDTA, 1 PMSF, pH 7.6) supplemented with the protease inhibitor cocktail. Insolubilized materials were removed by centrifugation at 4 °C. Solubilized cell lysates were incubated overnight at 4 °C with streptavidin-agarose beads (Thermo Scientific, Waltham, MA, USA). Beads were washed once in the lysis buffer, followed by twice in a high-salt buffer ((in mm) 500 NaCl, 5 EDTA, 50 Tris-HCl, pH7.6, 0.1% Triton X-100) and once in a low-salt buffer ((in mm) 2 EDTA, 10 Tris-HCl, pH7.6, 0.1% Triton X-100). Biotin-streptavidin complexes were eluted from the beads by heating at 70 °C for 5 min in the Laemmli sample buffer. 

For quantitative analyses, cell lysates from biotinylated intact cells were subject to either direct immunoblotting analyses (*Total*) or streptavidin pull-down prior to immunoblotting (*Surface*). Specifically, during sample loading for SDS-PAGE, the amount of lysates loaded in the *Total* lane represents about 8% of that used for streptavidin pull-down and thereafter loading in the *Surface* lane. For both *Total* and *Surface* signals, the protein density was standardized as the ratio to the cognate *Total* loading control. Standardized values were normalized with respect to those of the corresponding control group.

### 2.11. Statistical Analyses

All values were presented as mean ± SEM. Based on the assumption of normality and homogeneity of variance, the significance of the difference between two means was tested using the Student’s *t* test, whereas means from multiple groups were compared using the one-way ANOVA analysis, followed by post hoc analysis with the Bonferroni *t* test. Power analyses for statistical data are shown in Table S1. All statistical analyses were performed with Origin 7.0 (Microcal Software, Northampton, MA, USA).

## 3. Results

### 3.1. Proteasomal Degradation of ClC-2 is Mediated by Cullin 4 E3 Ubiquitin Ligase 

**Figure 1:**
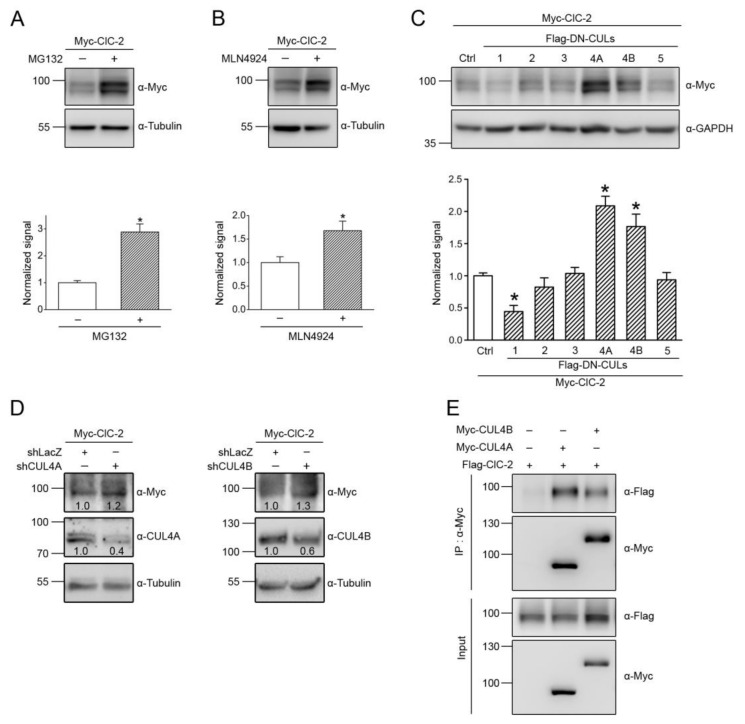
CUL4 mediates the protein degradation of ClC-2 channels. Myc- or Flag-tagged mouse ClC-2 channels (Myc-ClC-2, Flag-ClC-2) were overexpressed in HEK293T cells. (**A**,**B**) (*Upper panels*) Representative immunoblots showing the effect of 24-h treatment of 10 μM MG132 (in 0.1% DMSO) (**A**) or 10 μM MLN4924 (in 0.1% DMSO) (**B**) on the protein expression of Myc-ClC-2. The molecular weight markers (in kilodaltons) and immunoblotting antibodies (α-Myc and α-Tubulin) are labeled to the left and right, respectively. Tubulin was used as the loading control. (*Lower panels*) Quantification of relative ClC-2 protein levels. Protein density was standardized as the ratio of the ClC-2 signal to the cognate tubulin signal. Values from the MG-132-treated or the MLN4924-treated groups (*hatched bars*) were then normalized to those for the corresponding control (*clear bars*). Asterisks denote a significant difference from the control (*, *t* test: *p* < 0.05; *n* = 6–12). (**C**) (*Upper panel*) Representative immunoblots illustrating the effect of co-expressing Flag-tagged dominant-negative cullins (Flag-DN-CULs) on Myc-ClC-2. Co-expression with the Flag vector was used as the control experiment. GAPDH was used as the loading control. (*Lower panel*) Quantification of relative ClC-2 protein levels in the presence of various Flag-DN-CUL constructs (*, *t* test: *p* < 0.05; *n* = 7–16). (**D**) Representative immunoblots showing the effect of shRNA knockdown of endogenous CUL4A/B (shCUL4A/B) in HEK293T cells on Myc-ClC-2. Infection with shLacZ was used as the control experiment. Tubulin was used as the loading control. The numbers on the immunoblot denote the relative ClC-2 protein levels. (**E**) Co-immunoprecipitation of Flag-ClC-2 and Myc-CUL4A/B. Cells were incubated in 10 μM MG132 for 24 h before being solubilized. Co-expression with the Myc vector was used as the control experiment. Cell lysates were immunoprecipitated (IP) with α-Myc, followed by immunoblotting (IB) of the immunoprecipitates with α-Myc or the anti-Flag antibody (α-Flag). Corresponding expression levels of ClC-2 and CUL4A/B in the lysates are shown in the *Input* lane. In all cases hereafter, input represents about 10% of the total protein used for immunoprecipitation.

We began by investigating whether proteasomal degradation contributes to the proteolytic mechanism of mouse ClC-2 proteins overexpressed in HEK293T cells. The peptide aldehyde MG132 is an effective proteasome inhibitor suitable for addressing the biochemical role of proteasomal degradation [31,32]. As depicted in Figure 1A, treatment with 10 μM MG132 for 24 h resulted in almost a 3-fold increase in the ClC-2 protein level, suggesting that ClC-2 is subject to proteasomal degradation in HEK293T cells.

In the next line of experiments, we attempted to identify the E3 ubiquitin ligase responsible for ClC-2 degradation. To date, over 600 distinct E3 ligases are estimated to be expressed in human cells [33,34]. The cullin-really interesting new gene (RING) ligase, comprising more than 200 different members, constitutes the largest subfamily of human E3 ubiquitin ligases [35,36,37]. To test the hypothesis that cullin RING ligases may regulate ClC-2 protein expression, we employed a potent inhibitor of cullin RING E3 ligases, MLN4924 [38,39]. As illustrated in Figure 1B, the 24-h treatment with 10 μM MLN4924 induced a significant enhancement in the ClC-2 protein signal, consistent with the idea that cullin-RING ligases may contribute to ClC-2 protein degradation.

Cullin RING E3 ligases are multi-subunit protein complexes, consisting of the scaffold protein cullin that binds to both the RING-containing catalytic component and the substrate-binding component [35,36,37]. In human cells, there are several different subtypes of cullins (e.g., CUL1, CUL2, CUL3, CUL4A, CUL4B, and CUL5), each serving as the core of a subclass of cullin-RING ligases. In order to determine the specific cullin subtype in HEK293T cells that contributes to the regulation of ClC-2, we then studied the effects of various dominant-negative cullin constructs, which are cullin carboxyl-terminal truncation mutants that retain the substrate recognition capacity but lack the ubiquitin ligase function [40,41,42]. Figure 1C shows that only dominant-negative cullin 4A (DN-CUL4A) and 4B (DN-CUL4B) effectively increased the ClC-2 protein level. In addition, shRNA knockdown of endogenous CUL4A or CUL4B promoted ClC-2 protein expression in HEK293T cells (Figure 1D). To further investigate the potential interaction between CUL4A/B and ClC-2 channels, we performed an immunoprecipitation experiment by co-expressing ClC-2 and full-length CUL4A/B in HEK293T cells. As depicted in Figure 1E, both CUL4A and CUL4B were co-immunoprecipitated with ClC-2, consistent with the idea that CUL4A/B co-exists with ClC-2 in the same protein complex. Together, these observations support the notion that CUL4A and CUL4B are essential components of the E3 ubiquitin ligase complex promoting the degradation of ClC-2 channels.

### 3.2. Cereblon Serves as the Substrate Receptor Protein of CUL4 E3 Ligase Complex for ClC-2 Degradation 

**Figure 2:**
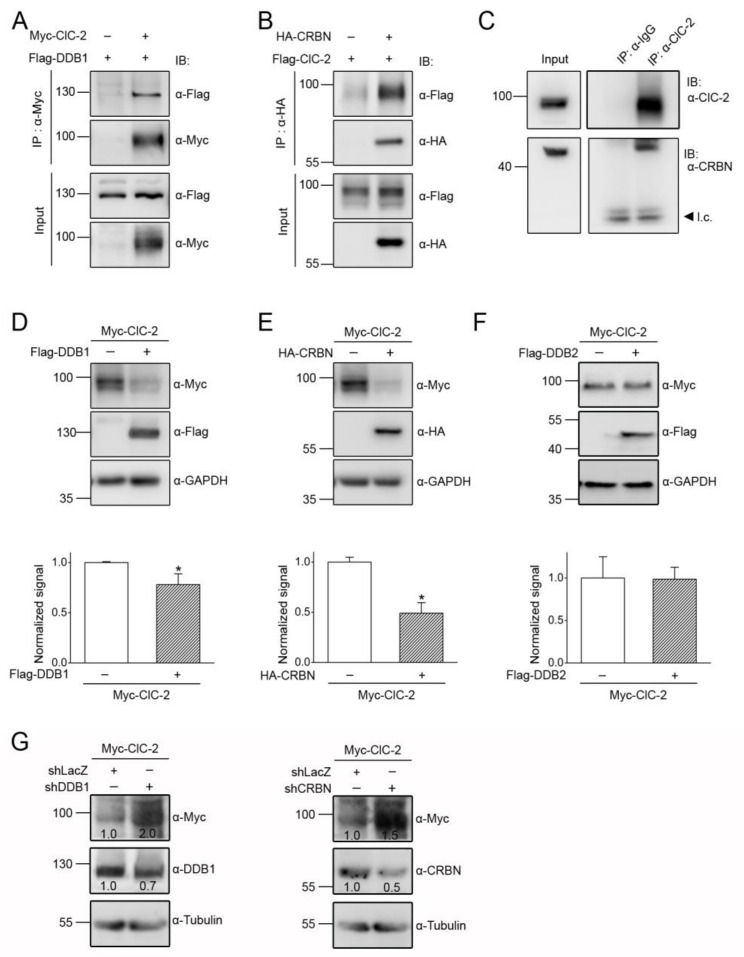
DDB1 and CRBN enhance ClC-2 protein degradation. (****A**,**B****) Co-immunoprecipitation of Myc-ClC-2 and Flag-DDB1 (**A**), as well as of Flag-ClC-2 and HA-tagged CRBN (HA-CRBN) (**B**), in HEK293T cells. Flag-DDB1 and HA-CRBN were recognized with α-Flag and the anti-HA antibody (α-HA), respectively. (**C**) Interaction of endogenous CRBN with ClC-2 in the rat brain. Whole brain lysates were immunoprecipitated with the anti-ClC-2 antibody (α-ClC-2), followed by immunoblotting of the immunoprecipitates with α-ClC-2 or the anti-CRBN antibody (α-CRBN). Co-immunoprecipitation of CRBN was achieved by using α-ClC-2 but not by rabbit IgG (α-IgG). l.c.: IgG light chain. (***D***–***E****) (Upper panels*) Representative immunoblots showing the effect of Flag-DDB1 (**D**) or HA-CRBN (**E**) co-expression on the Myc-ClC-2 protein level. Co-expression with the Flag or the HA vector was used as the control experiment. (*Lower panels*) Quantification of the relative ClC-2 protein levels. Values from the DDB1 or the CRBN co-expression groups (*hatched bars*) were normalized to those for the corresponding vector control (*clear bars*) (*, *t* test: *p* < 0.05; *n* = 7–10). (**F**) (*Upper panel*) Representative immunoblots showing the lack of an effect of Flag-DDB2 co-expression on the Myc-ClC-2 protein level. Co-expression with the Flag vector was used as the control experiment. (*Lower panel*) Quantification of relative ClC-2 protein levels (*t* test: *p* > 0.05; *n* = 3). (**G**) Representative immunoblots showing the effect of shRNA knockdown of endogenous DDB1 (shDDB1) or CRBN (shCRBN) in HEK293T cells on Myc-ClC-2. Infection with shLacZ was used as the control experiment. The numbers on the immunoblot denote the relative ClC-2 protein levels.

The substrate-recognition component of most cullin E3 ligases consists of an adaptor protein that directly interacts with the scaffold protein cullin, as well as a substrate receptor protein that determines the substrate specificity of a given cullin E3 ligase complex [35,36,37]. More than 90 different CUL4A/B E3 ubiquitin ligase complexes have been identified in mammals, and a canonical adaptor protein is damage-specific DNA binding protein 1 (DDB1), present in about 60 CUL4 E3 ligase complexes [43,44,45]. To test the hypothesis that DDB1 may serve as the adaptor protein mediating CUL4A/B regulation of ClC-2 channels, we co-expressed ClC-2 and DDB1 in HEK293T cells. Figure 2A illustrates that DDB1 was efficiently co-immunoprecipitated with CLC-2, consistent with the presence of a protein complex consisting of CUL4A/B, DDB1, and ClC-2.

We then went on to address the molecular nature of the substrate receptor linking CUL4A/B-DDB1 with ClC-2. Over 50 proteins have been suggested to serve as potential substrate receptors for CUL4A/B-DDB1 [45,46,47], and one of the newly identified substrate receptors is cereblon (CRBN) [48,49]. Interestingly, in vitro translated CRBN was previously shown to physically interact with the carboxyl terminal region of ClC-2 [50]. To determine whether CRBN and ClC-2 may form a protein complex in their native conformations, we co-expressed CRBN and ClC-2 in HEK293T cells. Figure 2B demonstrates that CRBN was co-immunoprecipitated with ClC-2. Moreover, we employed rat brain lysates to confirm that endogenous CRBN and ClC-2 in neurons co-exist in the same protein complex as well (Figure 2C).

**Figure 3:**
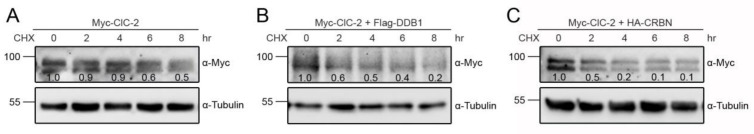
DDB1 and CRBN reduce ClC-2 protein stability. Representative immunoblots showing the effect of vector (**A**), DDB1 (**B**), or CRBN (**C**) co-expression on ClC-2 protein turnover kinetics in HEK293T cells. Transfected cells were subject to different treatment durations (0 to 8 h) of the protein synthesis inhibitor cycloheximide (CHX). Protein densities were normalized to those of corresponding no-treatment controls at 0 h. The numbers on the immunoblot denote relative ClC-2 protein levels.

To directly test whether DDB1 and CRBN may contribute to protein degradation of ClC-2, we studied the effect of DDB1/CRBN overexpression on ClC-2 protein level in HEK293T cells. Figure 2D,E show that the total ClC-2 protein level was significantly reduced in the presence of DDB1 and CRBN, respectively. In contrast, DDB2, a well-known substrate receptor for CUL4-DDB1 [45], failed to discernibly affect ClC-2 protein expression (Figure 2F). Conversely, shRNA knockdown of endogenous DDB1 or CRBN promoted ClC-2 protein expression in HEK293T cells (Figure 2G). Furthermore, by performing the cycloheximide chase analysis, we demonstrated that co-expression with DDB1 or CRBN leads to a substantial decrease in ClC-2 protein stability (Figure 3). Together, these results support the notion that CUL4 regulates ClC-2 protein degradation via the adaptor protein DDB1 and the substrate receptor protein CRBN.

### 3.3. CUL4 E3 Ligase Mediates Polyubiquitinaion of ClC-2 

**Figure 4:**
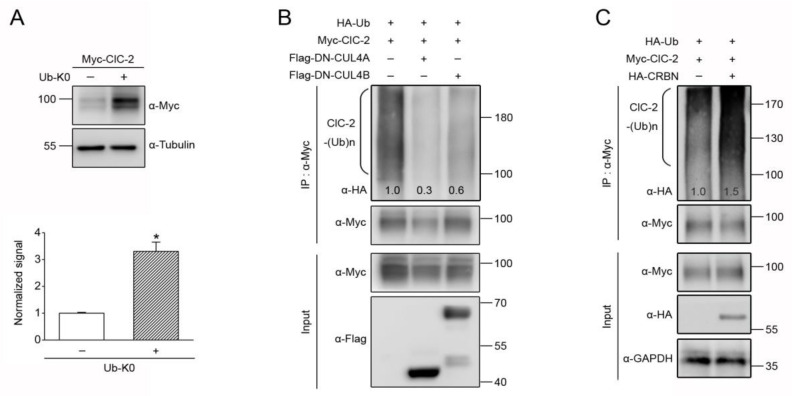
CUL4 E3 ligase promotes polyubiquitination of ClC-2 channels. (**A**) (*Upper panel*) Representative immunoblots showing the effect of the 24-h treatment of HA-tagged lysine-less ubiquitin (HA-Ub-K0) co-expression on the protein expression of Myc-ClC-2 in HEK293T cells. (*Lower panel*) Quantification of the relative ClC-2 protein levels. Protein density was standardized as the ratio of the ClC-2 signal to the cognate tubulin signal. Value from the Ub-K0 co-expression group (*hatched bar*) was then normalized to those for the vector control (*clear bar*). Asterisks denote a significant difference from the control (*, *t* test: *p* < 0.05; *n* = 5). (****B**,**C****) Representative immunoblots showing the effect of Flag-DN-CUL4A/B (**B**) or HA-CRBN (**C**) co-expression on ClC-2 polyubiquitination (ClC-2-(Ub)n) by HA-tagged ubiquitin (HA-Ub). Co-expression with the Flag/HA vector was used as the control experiment. The numbers shown on the immunoblot denote densitometric quantification of the relative ClC-2 ubiquitination with respect to the vector control. Cell lysates were immunoprecipitated (IP) with α-Myc/Flag, and protein ubiquitination was recognized by immunoblotting the immunoprecipitates with α-HA.

Prior to being degraded by proteasomes, proteins usually go through an ubiquitination process that involves covalent linkage of lysine residues with either single/multiple monoubiquitins or polyubiquitin chains [51,52]. One approach to differentiate these two distinct ubiquitin linkages is to inhibit polyubiquitin chain elongation by overexpressing a lysine-less ubiquitin (Ub-K0) mutant in which all of the lysines are mutated to arginines [53,54]. If a given protein is subject to notable polyubiquitination before being recognized by proteasomes, the application of Ub-K0 will be expected to reduce its proteolysis. Figure 4A shows that co-expression with Ub-K0 leads to more than 3-fold increase in the steady-state level of ClC-2, suggesting that proteasomal degradation of ClC-2 is preceded by considerable polyubiquitination of the Cl^−^ channel. Moreover, ClC-2 polyubiquitination, as demonstrated by co-expressing ClC-2 with HA-tagged ubiquitin (HA-Ub) and the ensuing presence of HA-UB-conjugated, high-molecular-weight ClC-2 protein smear, was dramatically attenuated by DN-CUL4A/B (Figure 4B). In contrast, co-expression with CRBN resulted in a substantial enhancement of ClC-2 polyubiquitination (Figure 4C). Overall, the preceding results are consistent with the idea that the CUL4 E3 ubiquitin ligase complex promotes ClC-2 polyubiquitination, which leads to proteasomal degradation of the Cl^−^ channel.

### 3.4. CUL4 E3 Ligase Regulates Endogenous ClC-2 Degradation 

**Figure 5:**
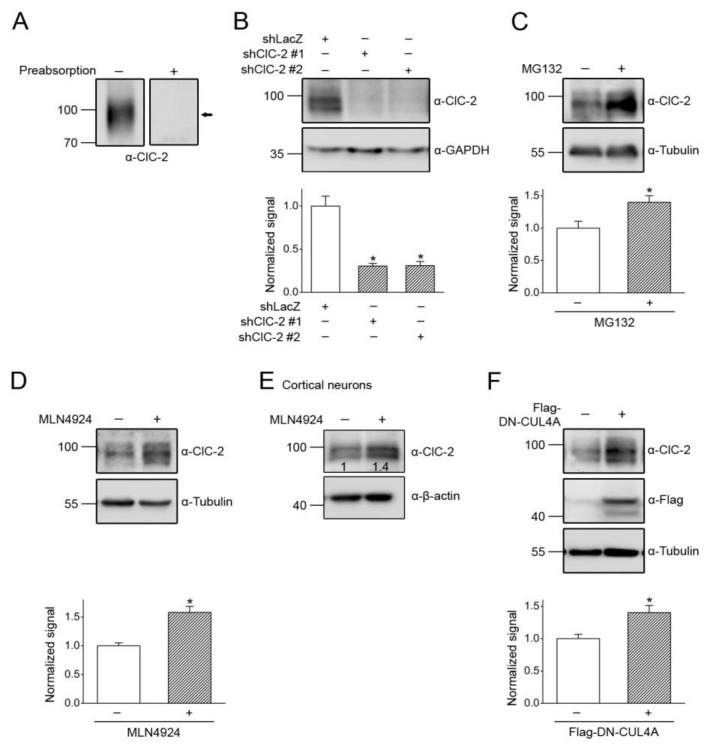
Regulation of endogenous ClC-2 expression by CUL4. (**A**) (*Left*) Endogenous expression of ClC-2 in the mouse testis. (*Right*) Verification of the specificity of α-ClC-2 in testes. Mouse ClC-2 protein detection was prevented by preabsorbing the immunoblot with a control antigen peptide. The protein band corresponding to mouse ClC-2 is highlighted with the black arrow. (**B**) (*Upper panel*) Representative immunoblot showing the effect of shRNA knockdown on the endogenous expression of ClC-2 in the mouse MA-10 Leydig cell. ClC-2 protein detection in MA-10 cells was prevented by infection with shRNA targeting specific mouse ClC-2 sequences (shClC-1 #1 and #2). shRNA targeting a LacZ sequence (shLacZ) was used as the control experiment. (*Lower panel*) Quantification of relative ClC-2 protein levels. Protein density was standardized as the ratio of the ClC-2 signal to the cognate GAPDH signal. Values from the shClC-2 groups (*hatched bars*) were then normalized to those for the corresponding shLacZ control (*clear bar*). Asterisk denotes a significant difference from the control (*, *t* test: *p* < 0.05; *n* = 4). (**C**) (*Upper panel*) Representative immunoblot showing the effect of the 24-h treatment of 10 μM MG132 on endogenous ClC-2 expression in MA-10 cells. (*Lower panel*) Quantification of the relative ClC-2 protein levels (*, *t* test: *p* < 0.05; *n* = 5). (**D**) (*Upper panel*) Representative immunoblot showing the effect of the 24-h treatment of 10 μM MLN4924 on endogenous ClC-2 expression in MA-10 cells. (*Lower panel*) Quantification of relative ClC-2 protein levels (*, *t* test: *p* < 0.05; *n* = 7). (**E**) Representative immunoblot showing the effect of MLN4924 treatment on endogenous ClC-2 expression in cultured rat cortical neurons. The numbers on the immunoblot denote the relative ClC-2 protein levels. (**F**) (*Upper panel*) Representative immunoblot showing the effect of Flag-DN-CUL4A overexpression on endogenous ClC-2 expression in MA-10 cells. Co-expression with the Flag vector was used as the control experiment. (*Lower panel*) Quantification of the relative ClC-2 protein levels (*, *t* test: *p* < 0.05; *n* = 7).

The foregoing data primarily focus on CUL4 E3 ligase regulation of mouse ClC-2 protein overexpressed in HEK293T cells. ClC-2 is profusely expressed in mouse testes (Figure 5A), and complete knockout of ClC-2 in mice leads to severe testicular degeneration, manifesting as a loss of mature sperms, abnormal Sertoli cells, and hyperplasia of Leydig cells [8,9]. In the following set of experiments, we aimed to verify our findings with endogenous ClC-2 channels in a physiologically relevant cell line, the MA-10 cell that is derived from mouse Leydig cells [55,56]. RNA interference with specific shRNA for ClC-2 confirmed the abundant expression of endogenous ClC-2 protein in MA-10 cells (Figure 5B). The ClC-2 protein level in MA-10 cells was robustly enhanced by treatment with the proteasome inhibitor MG132 (Figure 5C), indicating that endogenous ClC-2 is also subject to significant proteasomal degradation. Application of the cullin E3 ligase inhibitor MLN4924 led to prominent upregulation of the endogenous ClC-2 protein level in MA-10 cells (Figure 5D), as well as in primary cortical neurons (Figure 5E). Moreover, overexpression of DN-CUL4A resulted in about a 1.4-fold increase in the ClC-2 protein level in MA-10 cells (Figure 5F). 

**Figure 6:**
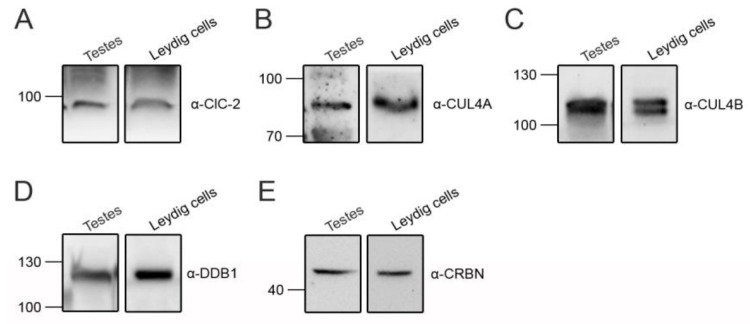
Endogenous expression of CUL4 E3 ligase in testes and Leydig cells. Representative immunoblots showing the endogenous expression of ClC-2 (**A**), CUL4A (**B**), CUL4B (**C**), DDB1 (**D**), and CRBN (**E**) in lysates prepared from mouse testes or cultured mouse Leydig cells. Approximately 30 µg of protein was loaded into each lane.

**Figure 7:**
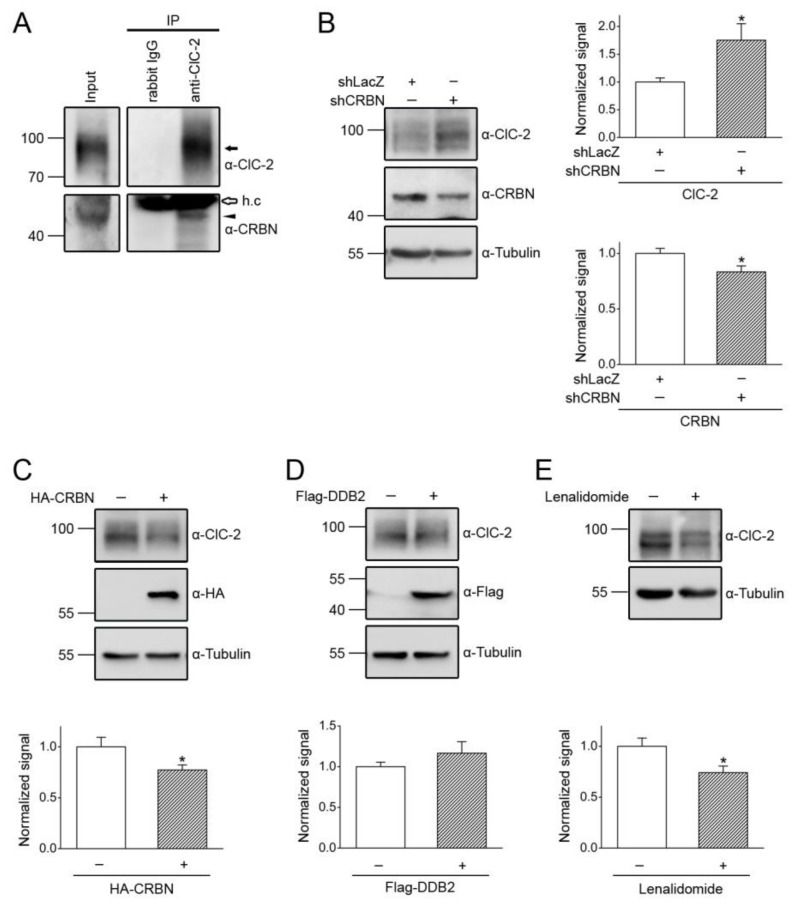
CRBN promotes degradation of endogenous ClC-2. (**A**) Co-immunoprecipitation of endogenous CRBN and ClC-2 in the mouse testis. Mouse testis lysates were immunoprecipitated with α-ClC-2 or the rabbit IgG. The protein bands corresponding to endogenous ClC-2 and CRBN are highlighted with the black arrow and the black arrowhead, respectively. The open arrow denotes the IgG heavy chain (h.c.). (**B**) (*Left panel*) Representative immunoblot showing the effect of shRNA knockdown of CRBN (shCRBN) on endogenous ClC-2 expression in the mouse MA-10 Leydig cell. Infection with shLacZ was used as the control experiment. (*Right panels*) Quantification of relative ClC-2 and CRBN protein levels. Protein density was standardized as the ratio of the ClC-2 signal to the cognate tubulin signal. Values from the shCRBN groups (*hatched bars*) were then normalized to those for the corresponding shLacZ control (*clear bar*). Asterisk denotes a significant difference from the control (*, *t* test: *p* < 0.05; *n* = 7). (**C**) (*Upper panel*) Representative immunoblot showing the effect of HA-CRBN overexpression on endogenous ClC-2 expression in MA-10 cells. Co-expression with the HA vector was used as the control experiment. (*Lower panel*) Quantification of relative ClC-2 protein levels (*, *t* test: *p* < 0.05; *n* = 7). (**D**) (*Upper panel*) Representative immunoblot showing the lack of an effect of Flag-DDB2 overexpression on endogenous ClC-2 expression in MA-10 cells. Co-expression with the Flag vector was used as the control experiment. (*Lower panel*) Quantification of relative ClC-2 protein levels (*t* test: *p* > 0.05; *n* = 9). (**E**) (*Upper panel*) Representative immunoblot showing the effect of the 24-h treatment of 10 μM lenalidomide (in 0.1% DMSO) on endogenous ClC-2 expression in MA-10 cells. (*Lower panel*) Quantification of relative ClC-2 protein levels (*, *t* test: *p* < 0.05; *n* = 6).

**Figure 8:**
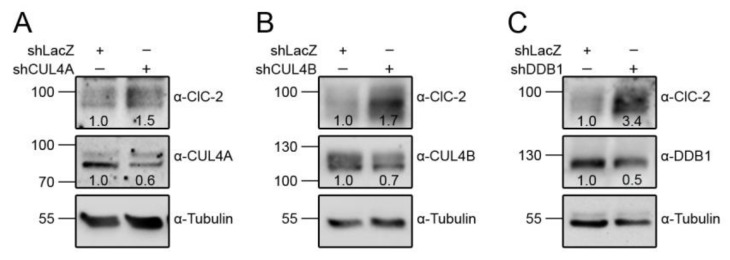
CUL4 and DDB1 promote the degradation of endogenous ClC-2. Representative immunoblots showing the effect of shRNA knockdown of CUL4A (shCUL4A) (**A**), CUL4B (shCUL4B) (**B**), or DDB1 (shCDDB1) (**C**) on endogenous ClC-2 expression in MA-10 cells. Infection with shLacZ was used as the control experiment. The numbers on the immunoblot denote the relative ClC-2 protein levels.

**Figure 9:**
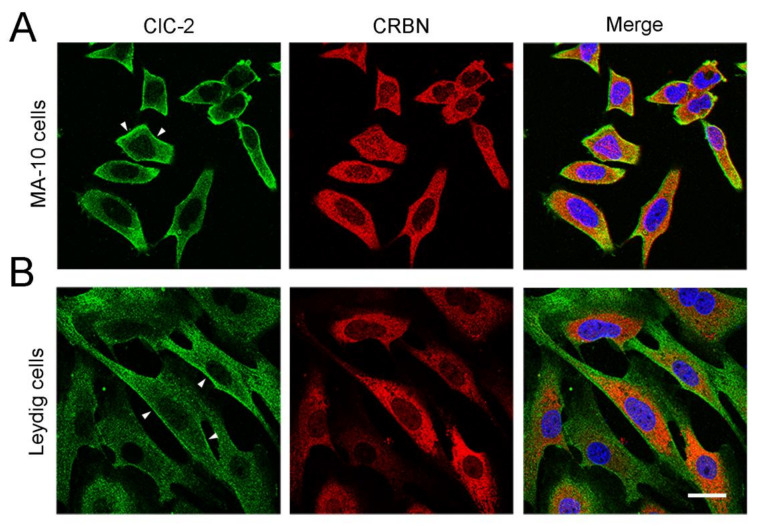
Subcellular localization of endogenous ClC-2 and CRBN. Representative confocal micrographs showing the immunofluorescence staining patterns of endogenous ClC-2 (*green*) and CRBN (*red*) in MA-10 cells (**A**) and cultured mouse Leydig cells (**B**). Merged images of ClC-2 and CRBN signals are shown in the rightmost panels, where cells were also stained with DAPI (*blue*) as a nuclear counterstain. Fixed cells were stained with the indicated antibodies under the permeabilized configuration. Arrowheads denote plasma membrane localization of ClC-2. Scale bar = 25 μm. Data are representative of three independent experiments.

CUL4A/B, DDB1, and CRBN are also abundantly expressed in mouse testes and Leydig cells (Figure 6). We then explored the role of the CUL4-DDB1 substrate receptor protein CRBN in endogenous ClC-2 proteostasis. The co-immunoprecipitation result in Figure 7A suggests that, in mouse testes, endogenous ClC-2 and CRBN co-exist in the same protein complex. Moreover, in MA-10 cells, shRNA knockdown of endogenous CRBN promoted ClC-2 protein expression (Figure 7B), whereas CRBN overexpression downregulated the ClC-2 protein level (Figure 7C). In contrast, overexpression of another CUL4-DDB1 substrate receptor protein, DDB2, did not detectably affect endogenous ClC-2 protein expression in MA-10 cells (Figure 7D). Several CRBN-targeting immunomodulatory drugs have recently been shown to be effective for treating multiple myeloma. For example, lenalidomide specifically binds to CRBN and promotes CUL4-mediated degradation of transcription factors implicated in the pathogenesis of myeloma [57,58,59]. Importantly, treatment with lenalidomide substantially suppressed the ClC-2 protein level in MA-10 cells (Figure 7E). Consistent with the regulatory role of CRBN, shRNA knockdown of endogenous CUL4A/B or DDB1 promoted ClC-2 protein expression in MA-10 cells (Figure 8). Additionally, immunofluorescence analyses in both MA-10 cells and mouse Leydig cells (Figure 9) indicated that ClC-2 is abundantly present at the plasma membrane and that a notable fraction of ClC-2 is also present in the cytoplasm and partially co-localizes with CRBN, further supporting the notion that the CUL4-DDB1-CRBN E3 ligase complex mediates ClC-2 protein degradation in mouse Leydig cells.

### 3.5. Correction of Disease-Associated ClC-2 Proteostasis Anomaly by Modifying CUL4 E3 Ligase Activity

Previous biochemical analyses have revealed that leukodystrophy-causing *CLCN2* mutations are associated with enhanced protein degeneration of ClC-2 [16,18]. Nevertheless, it is still an open question whether aldosteronism-causing *CLCN2* mutations may also affect ClC-2 proteostasis. To address this issue, we studied two disease-causing human ClC-2 mutations: The aldosteronism-associated G24D mutation (G32D in mouse ClC-2) and the leukodystrophy-associated G503R mutation (G511R in mouse ClC-2) [14,18]. 

**Figure 10:**
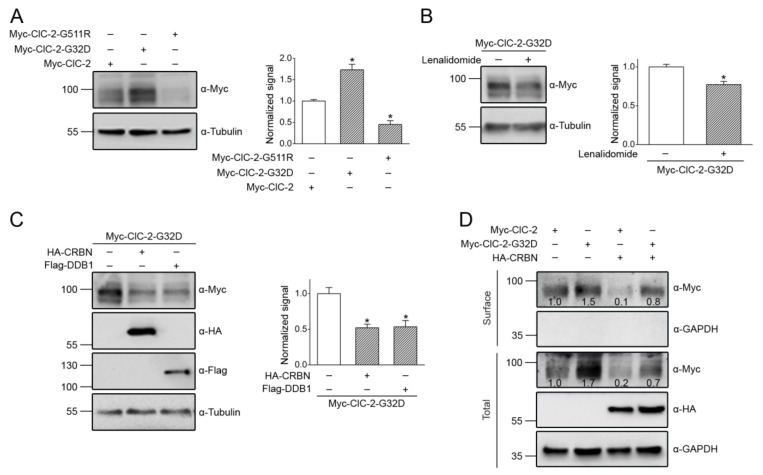
Aldosteronism-associated gain-of-function alteration in ClC-2 proteostasis. (**A**) (*Upper panel*) Representative immunoblot comparing the protein expression of Myc-ClC-2 WT, aldosteronism-related G32D mutant, and leukodystrophy-related G511R mutant overexpressed in HEK293T cells. (*Lower panel*) Quantification of relative ClC-2 protein levels. Protein density was standardized as the ratio of the ClC-2 signal to the cognate tubulin signal. Values from the mutant groups (*hatched bars*) were then normalized to those for the corresponding WT control (*clear bars*). Asterisks denote a significant difference from the WT (*, *t* test: *p* < 0.05; *n* = 7–9). (**B**) (*Upper panel*) Representative immunoblot showing the effect of the 24-h treatment of 10 μM lenalidomide on the Myc-ClC-2 G32D mutant. (*Lower panel*) Quantification of the relative ClC-2 G32D protein levels (*, *t* test: *p* < 0.05; *n* = 7). (**C**) (*Upper panel*) Representative immunoblot showing the effect of HA-CRBN or Flag-DDB1 co-expression on the Myc-ClC-2 G32D mutant. (*Lower panel*) Quantification of the relative ClC-2 G32D protein levels (*, *t* test: *p* < 0.05; *n* = 4–6). (**D**) Representative immunoblot showing the enhanced surface expression of the Myc-ClC-2 G32D mutant, as well as its reduction by CRBN co-expression. Cell lysates from surface-biotinylated intact HEK293T cells were subject to either direct immunoblotting analyses (*Total*) or streptavidin pull-down prior to immunoblotting (*Surface*). The numbers on the immunoblot denote the relative ClC-2 protein levels.

Figure 10A demonstrates that, consistent with the previous finding that the leukodystrophy-causing human G503R mutant displays defective ClC-2 proteostasis [18], the total protein level of the equivalent mouse ClC-2 G511R mutant was reduced by more than 50%. In contrast, the mouse ClC-2 G32D mutant, equivalent to the aldosteronism-causing human ClC-2 G24D mutation, was associated with a dramatic enhancement in the total protein level by more than 70% (Figure 10A), reminiscent of the gain-of-function change in the functional expression of the mutant human ClC-2 channel [14]. These observations strongly suggest that the pathophysiological mechanism of the two channelopathies may entail opposite alterations in ClC-2 proteostasis.

Given that CUL4-DDB1-CRBN plays a critical role in regulating ClC-2 proteostasis, the next question we asked was whether the foregoing disease-associated alterations in ClC-2 proteostasis may be corrected by modulating CUL4 E3 ligase activity. Consistent with its effect on downregulating endogenous ClC-2 protein level in MA-10 cells (Figure 7E), promoting CUL4 ligase activity with the CRBN-targeting lenalidomide resulted in a notable decrease in the total protein level of the gain-of-function mouse ClC-2 G32D mutant in HEK293T cells (Figure 10B). Likewise, overexpression of either DDB1 or CRBN downregulated the protein expression of the aldosteronism-associated ClC-2 mutant by about 50% (Figure 10C). The gain-of-function proteostasis change did not detectably affect the membrane trafficking property of ClC-2 G32D, since cell surface protein expression of the mutant Cl^−^ channel appeared to be proportionately enhanced (Figure 10D). Furthermore, CRBN co-expression effectively attenuated the surface protein level of both ClC-2 WT and the G32D mutant (Figure 10D).

**Figure 11:**
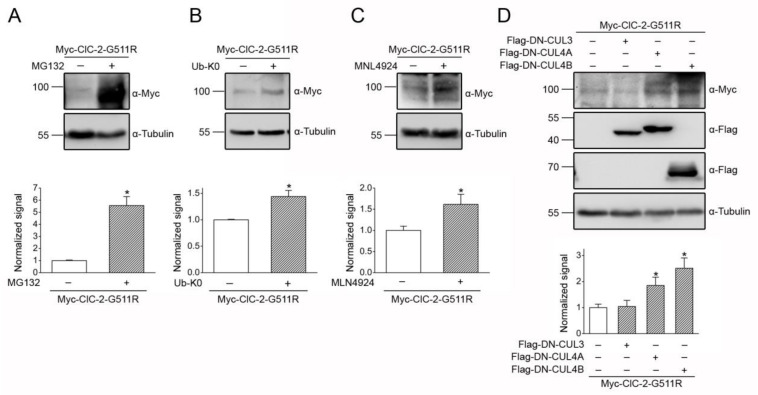
CUL4 E3 ligase contributes to leukodystrophy-associated defective ClC-2 proteostasis. (**A**) (*Upper panel*) Representative immunoblot showing the effect of the 24-h treatment of 10 μM MG132 on the Myc-ClC-2 G511R mutant in HEK293T cells. (*Lower panel*) Quantification of the relative ClC-2 G511R protein levels (*, *t* test: *p* < 0.05; *n* = 6). (**B**) (*Upper panel*) Representative immunoblot showing the effect of the 24-h treatment of HA-UB-K0 co-expression on the Myc-ClC-2 G511R mutant. (*Lower panel*) Quantification of relative ClC-2 G511R protein levels (*, *t* test: *p* < 0.05; *n* = 3). (**C**) (*Upper panel*) Representative immunoblot showing the effect of the 24-h treatment of 10 μM MLN4924 on the Myc-ClC-2 G511R mutant. (*Lower panel*) Quantification of the relative ClC-2 G511R protein levels (*, *t* test: *p* < 0.05; *n* = 4). (**D**) (*Upper panel*) Representative immunoblot showing the effect of Flag-DN-CUL3/CUL4A/CUL4B co-expression on the Myc-ClC-2 G511R mutant. (*Lower panel*) Quantification of the relative ClC-2 G511R protein levels (*, *t* test: *p* < 0.05; *n* = 6).

On the other hand, treatment with the proteasome inhibitor MG132 led to a more than 5-fold increase in the total protein level of the loss-of-function mouse ClC-2 G511R mutant (Figure 11A). Similarly, suppressing ClC-2 degradation by blocking polyubiquitination via overexpression of Ub-K0 (Figure 11B), as well as by inhibition of cullin RING E3 ligase function with MLN4924 (Figure 11C), markedly promoted the protein expression of the G511R mutant. Moreover, Figure 11D highlights that impeding the endogenous CUL4 function in HEK293T cells by overexpressing the dominant-negative construct DN-CUL4A or DN-CUL4B effectively enhanced the protein level of the leukodystrophy-associated ClC-2 mutant. In contrast, obstructing the endogenous CUL3 function with DN-CUL3 failed to measurably affect the defective proteostasis of the ClC-2 mutant (Figure 11D). Taken together, our data support the notion that modifying CUL4 E3 ligase activity may effectively rescue disease-associated ClC-2 proteostasis abnormality. 

## 4. Discussion

**Figure 12:**
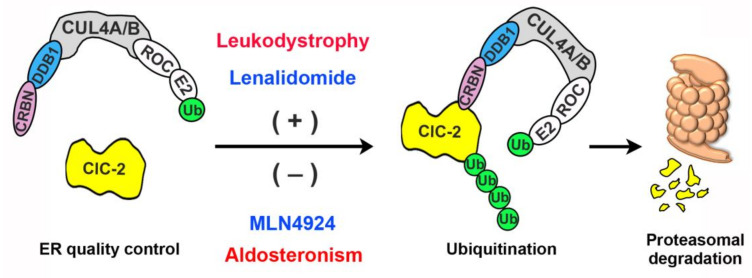
Schematic model of endoplasmic reticulum (ER)-associated degradation of ClC-2. In this schematic diagram of the regulation of ClC-2 proteostasis by ER quality control, the scaffold protein CUL4A/B forms a protein complex with the adaptor protein DDB1 and the substrate receptor protein CRBN. CUL4A/B also interacts with the RING-finger protein ROC, which in turn recruits the E2 ubiquitin conjugating enzyme (E2) that transfers ubiquitin (Ub) for covalent linkage to a substrate protein. We propose that, through the direct interaction between CRBN and ClC-2, the CUL4A/B-DDB1-CRBN E3 ubiquitin ligase complex catalyzes the ubiquitination of misfolded ClC-2 proteins. Ubiquitinated ClC-2 is subsequently targeted for proteasomal degradation. Loss-of-function, leukodystrophy-causing mutations may instigate substantial protein misfolding, leading to enhanced degradation of mutant ClC-2 proteins. In contrast, gain-of-function aldosteronism-causing mutations appear to facilitate protein stability, thereby reducing proteasomal degradation of mutant ClC-2 channels. The CRBN-targeting immunomodulatory drug lenalidomide effectively promotes, whereas the cullin E3 ligase inhibitor MLN4924 significantly attenuates, proteasomal degradation of both ClC-2 WT and disease-associated mutant proteins.

In this study, we focused on the proteostasis mechanism of the ClC-2 Cl^−^ channel, whose gain-of-function and loss-of-function mutations have been associated with aldosteronism and leukodystrophy, respectively. Specifically, we aimed to elucidate the role of ER-associated proteasomal degradation in ClC-2 channelopathies. Serum and glucocorticoid inducible kinase isoforms SGK1-3 were previously suggested to modulate heterologous functional expression of the rat ClC-2 channel, presumably via phosphorylation of the E3 ubiquitin ligase Nedd4-2 localized at the cell surface [60]. It remains unclear, however, whether Nedd4-2 may directly interact with or regulate the protein expression of ClC-2. Importantly, the molecular machinery responsible for proteasomal degradation of ClC-2 channels has never been addressed. Herein, we provide multiple lines of biochemical evidence showing that, in both the heterologous expression system and native neuronal and testicular cells, ClC-2 co-exists in the same protein complex with CUL4, DDB1, and CRBN. Moreover, our data are consistent with the idea that CUL4-DDB1-CRBN promotes ubiquitination and degradation of ClC-2. As depicted in the ER-associated degradation model in Figure 12, the core architecture of the CUL4 RING E3 ubiquitin ligase complex comprises the scaffold protein CUL4A/B, the RING-finger protein ROC, and a cognate E2 ubiquitin conjugating enzyme [43,45]. We propose that, through the substrate-recognition component composed of DDB1 and CRBN, the CUL4 E3 protein complex recruits the ClC-2 channel for polyubiquitination, which leads to subsequent proteasomal degradation of the Cl^−^ channel. 

The ClC-2 channel is a member of the CLC channel/transporter superfamily that includes five different types of Cl^−^ channels: ClC-0, ClC-1, ClC-2, ClC-Ka, and ClC-Kb [5,61,62]. Despite sharing similar protein structures and ion-permeation mechanisms, these Cl^−^ channels exhibit distinct tissue-specific localization patterns and voltage-dependent channel gating properties. For example, in direct contrast to the universally expressed hyperpolarization-activated ClC-2 channel, the ClC-1 channel is specifically expressed in skeletal muscles and activated by membrane depolarization. Loss-of-function mutations in the human *CLCN1* gene that instigate defective gating and proteostasis of the ClC-1 channel have been associated with the hereditary muscle disease myotonia congenita [5,63]. Interestingly, we previously demonstrated that ClC-1 WT and disease-causing mutant proteins are also subject to polyubiquitination and degradation by the CUL4-DDB1-CRBN E3 ubiquitin ligase [64]. It remains an open question whether the CUL4 E3 ligase complex may mediate protein degradation of the other members of the CLC channel/transporter superfamily. 

Emerging evidence supports the association of many disease-causing mutant proteins with anomalous proteostasis [24,65,66]. In addition to altered voltage-dependent gating properties, leukodystrophy-causing *CLCN2* mutations lead to defective human ClC-2 proteostasis, manifesting as impaired protein stability and membrane trafficking [16,18]. Similarly, we demonstrated in the current study that a disease-associated equivalent mutant in mouse ClC-2 (G511R) also displays a drastically reduced protein level, further supporting the notion that ClC-2 proteins harboring leukodystrophy-causing mutations are prone to proteasomal degradation (Figure 12). Significantly, our biochemical analyses provide additional insight into the defective proteostasis of the ClC-2-G511R mutant. In ClC-2 WT, both MG132 treatment and Ub-K0 co-expression led to about a 3-fold increase in the protein level (Figure 1A and Figure 4A), consistent with the idea that the vast majority of the ClC-2 WT channel is subject to polyubiquitination prior to eventual proteasomal degradation. In comparison, the MG132 treatment resulted in a more than 5-fold increase in the protein level of ClC-2 G511R (Figure 11A), indicating that the loss-of-function mutant is associated with dramatically enhanced proteasomal degradation. Surprisingly, in direct contrast to the comparable effects of MG132 and Ub-K0 in boosting ClC-2 WT protein expression, suppression of polyubiquitination with Ub-K0 only upregulated the G511R protein level by about 1.4-fold (Figure 11B), suggesting that, for a substantial amount of the mutant channel, polyubiquitination may not be required for proteasomal degradation. The precise reason underlying this mismatch between the effect of MG132 treatment and Ub-K0 co-expression on rescuing the G511R protein level is currently unclear. This unexpected observation may nonetheless imply that the leukodystrophy-causing ClC-2 G511R mutant is endowed with a substantial protein folding defect that may trigger additional ER-associated proteasomal degradation mechanisms not commonly associated with the ClC-2 WT channel. 

Importantly, we also provide the first direct evidence indicating that an equivalent aldosteronism-causing mutant in mouse ClC-2 (G32D) is associated with a more than 70% increase in the total protein level, suggesting that this disease-causing mutation may affect the biochemical property of ClC-2 channels by promoting protein expression. This apparent gain in ClC-2 proteostasis is reminiscent of the gain-of-function phenotype of aldosteronism-causing human ClC-2 mutant channels that manifest as enhanced membrane Cl^−^ currents in aldosterone-producing adrenal glomerulosa cells [12,13,14]. We therefore propose that, in addition to promoting voltage-dependent channel activation, aldosteronism-causing *CLCN2* mutations may also facilitate ClC-2 protein folding and consequently reduce ER-associated degradation of the Cl^−^ channel (Figure 12). Further biochemical and biophysical investigations will be required to address the molecular mechanism underlying the opposite effects on ClC-2 proteostasis imposed by aldosteronism- and leukodystrophy-related mutations.

One of the current therapeutic strategies for human diseases associated with proteostasis impairment focuses on developing biological and chemical approaches effective in facilitating protein folding and optimizing the proteostasis capacity [65,67,68]. The CRBN-targeting immunomodulatory drug lenalidomide effectively promotes the degradation of many CRBN-binding substrate proteins [57,58,59], and is widely applied for the treatment of multiple myeloma [69,70]. Moreover, MLN4924, which prevents Nedd8 conjugation to the scaffold protein cullin and thereby inactivates cullin E3 ligase functions [38,39], is an emerging anticancer drug [71,72,73]. As summarized in the schematic model in Figure 12, we presented pharmacological evidence showing that the CRBN-activating lenalidomide and the CUL4-inhibiting MLN4924 promotes and attenuates, respectively, proteasomal degradation of WT/mutant ClC-2 proteins. These proof-of-concept observations are consistent with the idea that drugs targeting CUL4 E3 ubiquitin ligase activity may effectively rescue ClC-2 proteostasis anomaly. In addition, bortezomib, a 26S-proteasome inhibitor, has been clinically applied as an anticancer agent [74], and may be useful in rescuing the proteostasis defect of a schizophrenia-associated mutant potassium channel [75]. It will therefore be interesting to explore the therapeutic potential of lenalidomide and MLN4924/bortezomib in correcting ClC-2 proteostasis anomaly associated with aldosteronism and leukodystrophy, respectively. Furthermore, our discovery of the critical role of the CUL4-DDB1-CRBN E3 ligase complex may shed light on future elucidation of additional molecular machinery essential for maintaining ClC-2 proteostasis at the ER and the plasma membrane.

## Supplementary Materials

The following are available online at https://www.mdpi.com/2073-4409/9/6/1332/s1, Figure S1: Uncropped images of the immunoblots presented in the main figures, Table S1: Power analyses for the statistical data presented in the main figures.

## Abbreviations

Cl^−^	chloride
CRBN	cereblon
CUL	cullin
DDB	damage-specific DNA binding protein
D-PBS	Dulbecco’s phosphate buffered saline
DIV	days in vitro
DMEM	Dulbecco’s modified Eagle’s medium
DTT	dithiothreitol
ER	endoplasmic reticulum
FBS	Fetal bovine serum
GAPDH	glyceraldehyde-3-phosphate dehydrogenase
HEK	human embryonic kidney
IP	immunoprecipitation
MLC1	megalencephalic leukoencephalopathy with subcortical cysts 1
PBS	Phosphate buffered saline
PMSF	phenylmethylsulfonyl fluoride
RING	really interesting new gene
Ub	ubiquitin
Ub-K0	lysine-less ubiquitin
WT	wild-type.
