# Supplementary material for: CUL4-DDB1-CRBN E3 Ubiquitin Ligase Regulates Proteostasis of ClC-2 Chloride Channels: Implication for Aldosteronism and Leukodystrophy
Source: Cells. 2020 May 26;9(6):1332. doi: 10.3390/cells9061332 (PMC7348978; doi:10.3390/cells9061332)

Supplementary Material

CUL4-DDB1-CRBN E3 ubiquitin ligase regulates proteostasis of ClC-2 chloride channels: implication for aldosteronism and leukodystrophy

Ssu-Ju Fu, Meng-Chun Hu, Yi-Jheng Peng, Hsin-Yu Fang, Cheng-Tsung Hsiao,

Tsung-Yu Chen, Chung-Jiuan Jeng, and Chih-Yung Tang

Supplementary Table S1. Power analyses for the statistical data presented in the main figures.

Supplementary Figure S1. Uncropped images of the immunoblots presented in the main figures.

Supplementary Table S1. Power analyses for the statistical data presented in the main figures.

Fig. 1A

Two Sample Independent t-Test

t	DoF	P Value
-6.29041	10	0.00009

Power Analysis

Alpha	Total Sample Size	Power
0.05	12	0.99886

Fig. 1B

Two Sample Independent t-Test

t	DoF	P Value
-3.34548	22	0.00293

Power Analysis

Alpha	Total Sample Size	Power
0.05	24	0.89161

Fig. 1C

One-Way ANOVA

Source	DoF	Sum of Squares	Mean Square	F Value	P Value
Model	6	21.7004134	3.61673557	16.99584	0.00000
Error	60	12.7680705	0.212801176		

Power Analysis

Total

Alpha	Sample Size	Power
0.05	67	1.00000

Fig. 2D

Two Sample Independent t-Test

t	DoF	P Value
2.97363	18	0.00814

Power Analysis

Alpha	Total Sample Size	Power
0.05	20	0.80288

Fig. 2E

Two Sample Independent t-Test

t	DoF	P Value
4.8717	12	0.00038

Power Analysis

Alpha	Total Sample Size	Power
0.05	14	0.99022

Fig. 2F

Two Sample Independent t-Test

t	DoF	P Value
0.09489	4	0.92897

Power Analysis

Alpha	Total Sample Size	Power
0.05	6	0.05027

Fig. 4A

Two Sample Independent t-Test

t	DoF	P Value
-6.63852	8	0.00016

Power Analysis

Alpha	Total Sample Size	Power
0.05	10	0.99876

Fig. 5B

One-Way ANOVA

Source	DoF	Sum of Squares	Mean Square	F Value	P Value
Model	2	1.27886667	0.639433333	150.25849	0.00000
Error	9	0.0383000000	0.00425555556		

Power Analysis

Alpha	Total Sample Size	Power
0.05	12	1.00000

Fig. 5C

Two Sample Independent t-Test

t	DoF	P Value

-3.87966	8	0.00468

Power Analysis

Alpha	Total Sample Size	Power
0.05	10	0.92303

Fig. 5D

Two Sample Independent t-Test

t	DoF	P Value
-5.51817	12	0.00013

Power Analysis

Alpha	Total Sample Size	Power
0.05	14	0.99706

Fig. 5F

Two Sample Independent t-Test

t	DoF	P Value
-3.62144	12	0.00351

Power Analysis

Alpha	Total Sample Size	Power
0.05	14	0.91269

Fig. 7B

(ClC-2 level)

Two Sample Independent t-Test

t	DoF	P Value
-2.53797	12	0.02604

Power Analysis

Alpha	Total Sample Size	Power
0.05	14	0.63739

(CRBN level)

Two Sample Independent t-Test

t	DoF	P Value
3.10685	4	0.03598

Power Analysis

Alpha	Total Sample Size	Power
0.05	6	0.62325

Fig. 7C

Two Sample Independent t-Test

t	DoF	P Value
4.53116	12	0.00069

Power Analysis

Alpha	Total Sample Size	Power
0.05	14	0.98173

Fig. 7D

Two Sample Independent t-Test

t	DoF	P Value
-1.17778	16	0.25611

Power Analysis

Alpha	Total Sample Size	Power
0.05	18	0.18234

Fig. 7E

Two Sample Independent t-Test

t	DoF	P Value
4.44797	10	0.00124

Power Analysis

Alpha	Total Sample Size	Power
0.05	12	0.97467

Fig. 10A

One-Way ANOVA

Source	DoF	Sum of Squares	Mean Square	F Value	P Value
Model	2	6.62935873	3.31467937	48.35607	0.00000
Error	22	1.50804127	0.0685473304		

Power Analysis

Alpha	Total Sample Size	Power
0.05	25	1.00000

Fig. 10B

Two Sample Independent t-Test

t	DoF	P Value
5.68947	12	0.00010

Power Analysis

Alpha	Total Sample Size	Power
0.05	14	0.99785

Fig. 10C

One-Way ANOVA

Source	DoF	Sum of Squares	Mean Square	F Value	P Value
Model	2	6.62935873	3.31467937	48.35607	0.00000
Error	22	1.50804127	0.0685473304		

Power Analysis

Alpha	Total Sample Size	Power
0.05	25	1.00000

Fig. 11A

Two Sample Independent t-Test

t	DoF	P Value
-6.13141	10	0.00011

Power Analysis

Total

Alpha	Sample Size	Power
0.05	12	0.99853

Fig. 11B

Two Sample Independent t-Test

t	DoF	P Value
-3.81528	4	0.01885

Power Analysis

Alpha	Total Sample Size	Power
0.05	6	0.82260

Fig. 11C

Two Sample Independent t-Test

t	DoF	P Value
-2.57954	6	0.04180

Power Analysis

Alpha	Total Sample Size	Power
0.05	8	0.55178

Fig. 11D

One-Way ANOVA

Source	DoF	Sum of Squares	Mean Square	F Value	P Value
Model	3	9.25676061	3.08558687	7.29840	0.00210
Error	18	7.60996667	0.422775926		

Power Analysis

Alpha	Total Sample Size	Power
0.05	22	0.95389

Supplementary Figure S1. Uncropped images of the immunoblots presented in the main figures.

(related to Figure 1)

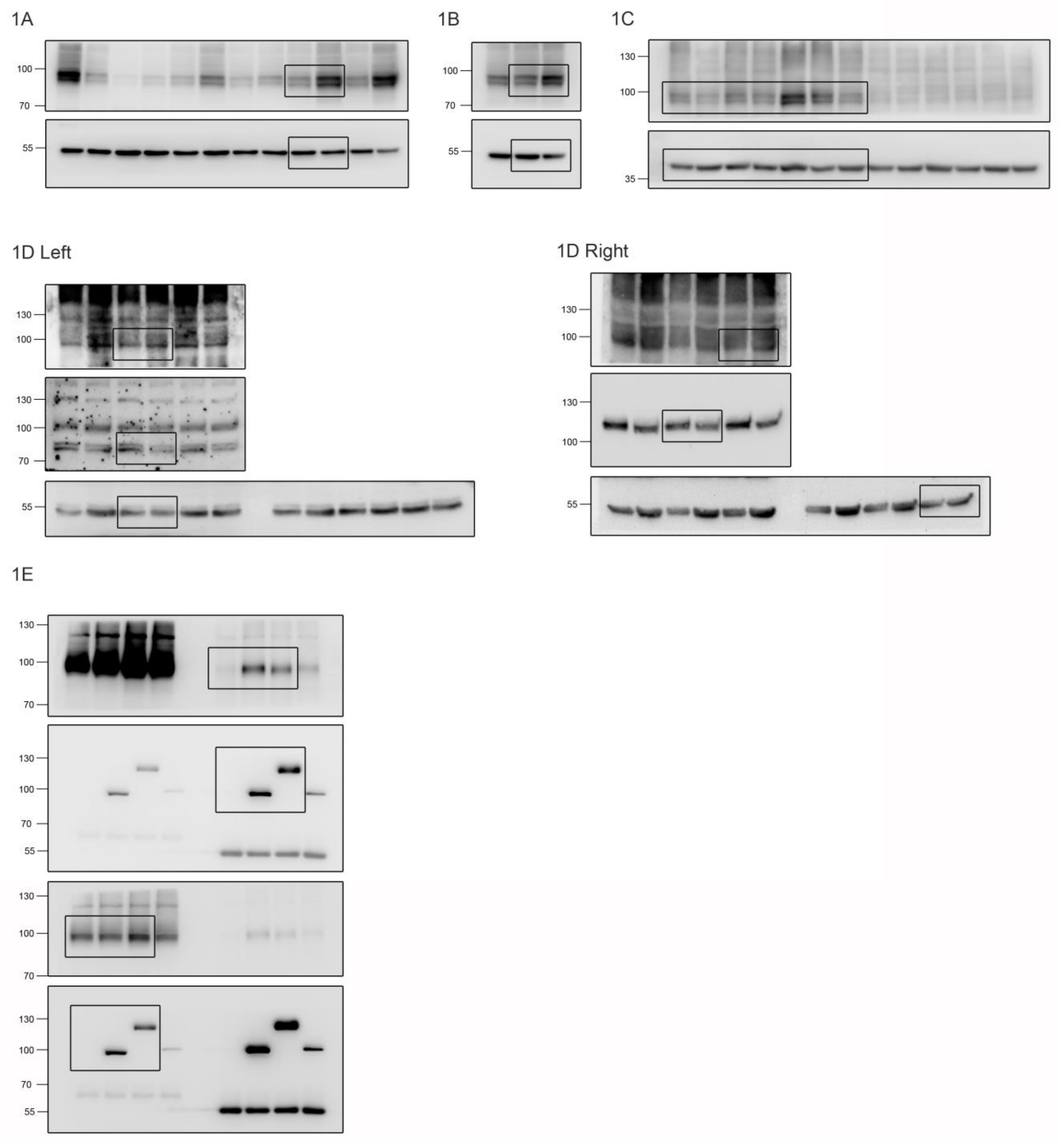

(related to Figure 2)

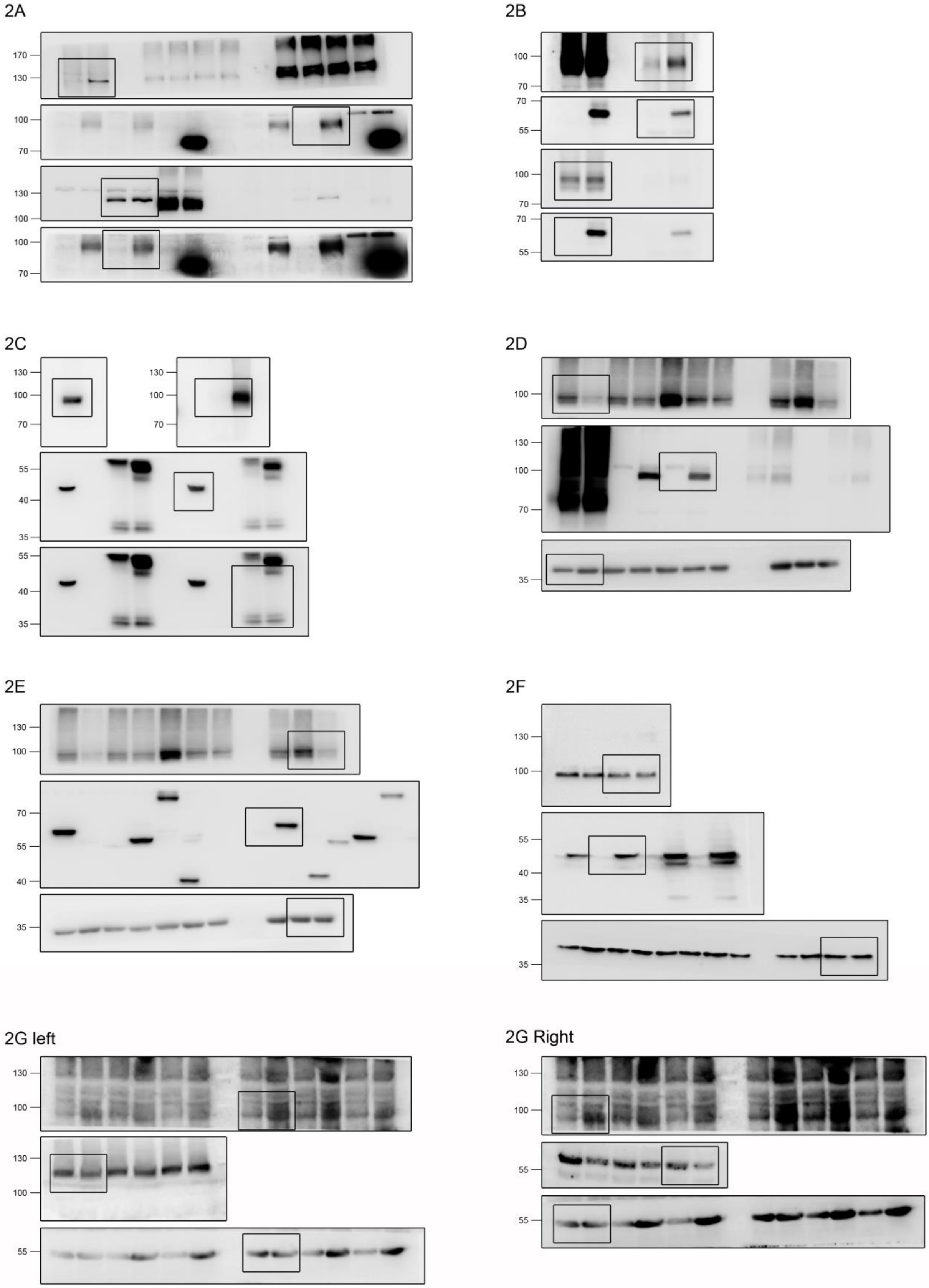

(related to Figure 3)

3A

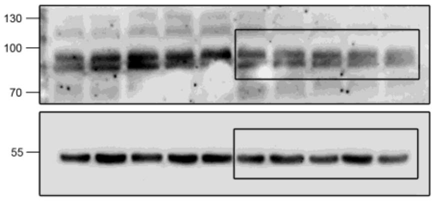

3B

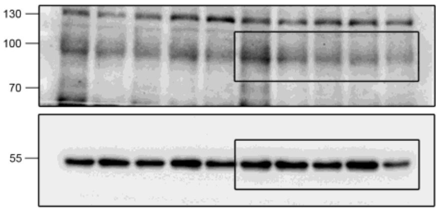

3C

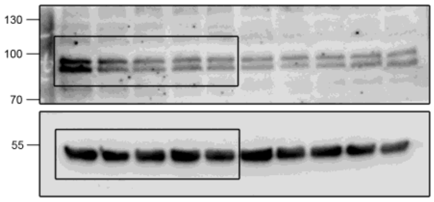

(related to Figure 4)

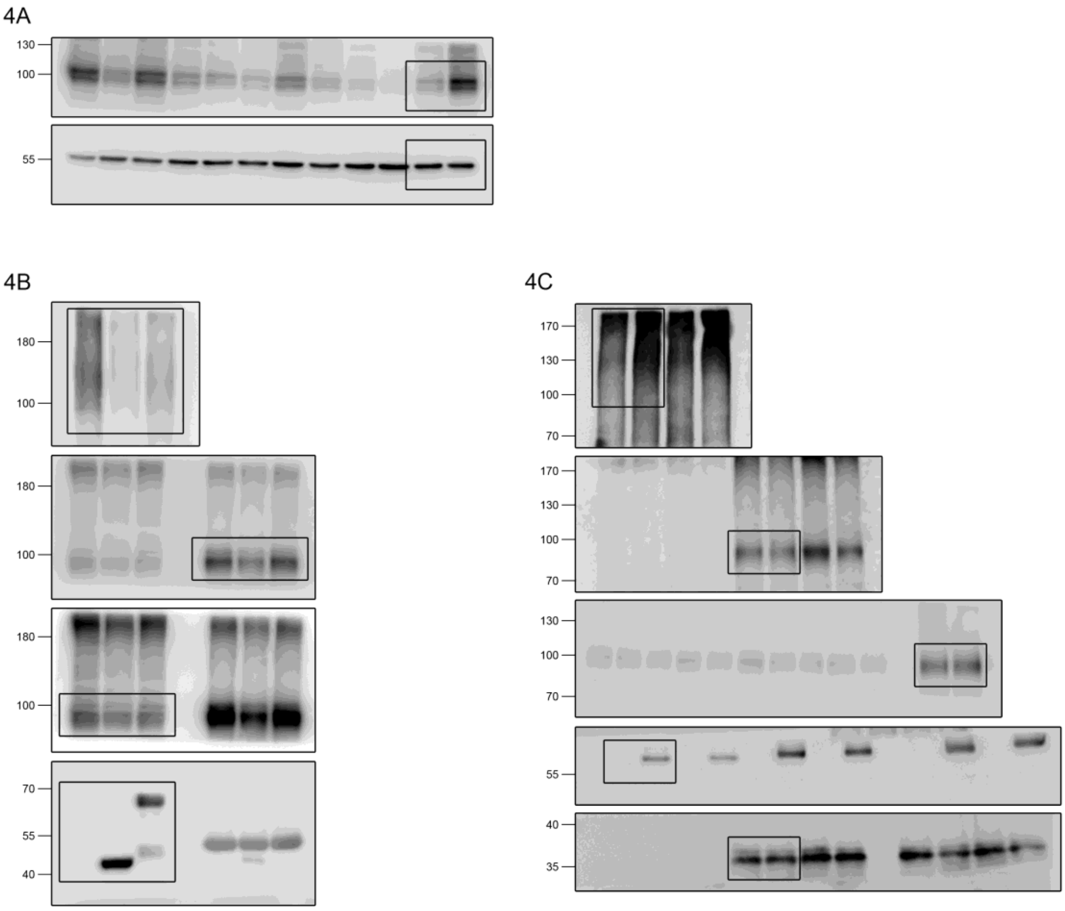

(related to Figure 5)

5A

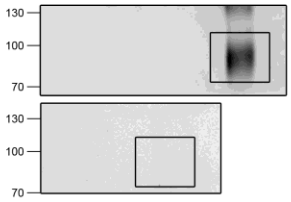

5B

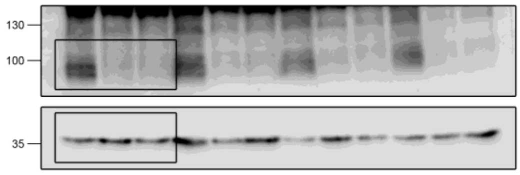

5C

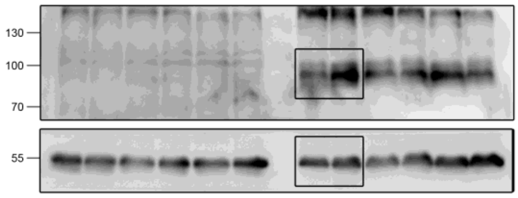

5D

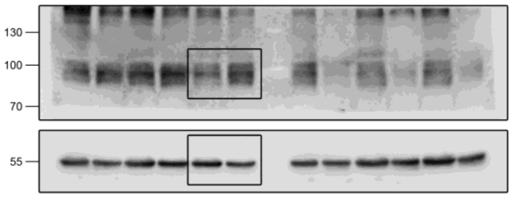

5E

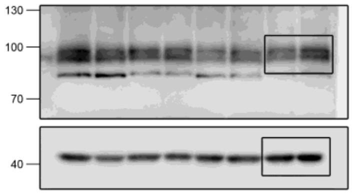

5F

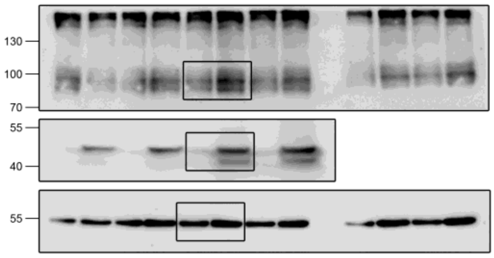

(related to Figure 6)

6A

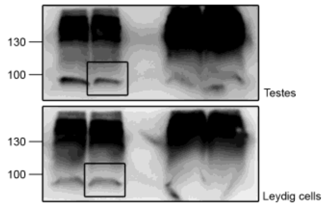

6B

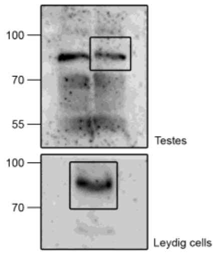

6C

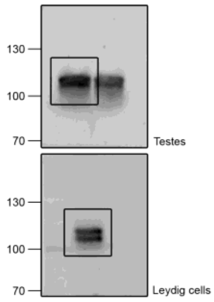

6D

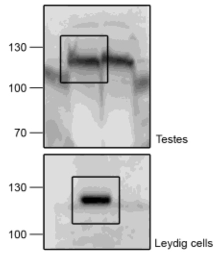

6E

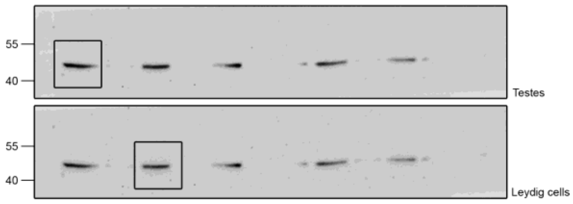

(related to Figure 7)

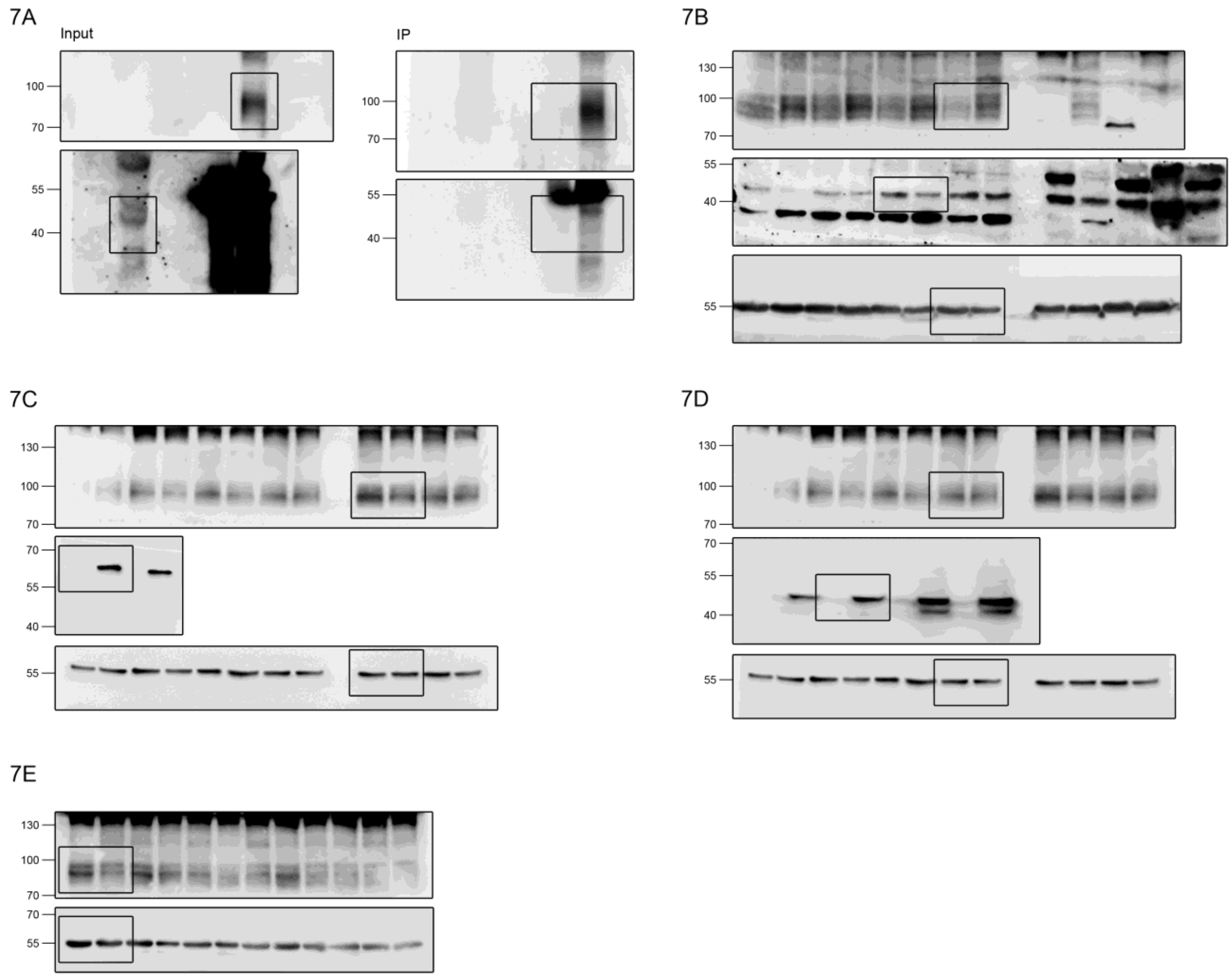

(related to Figure 8)

8A

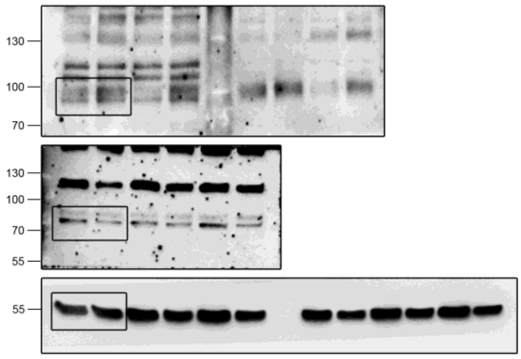

8B

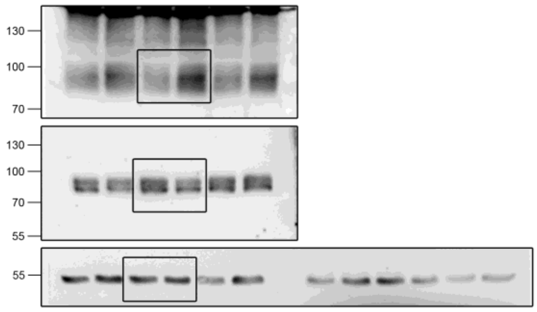

8C

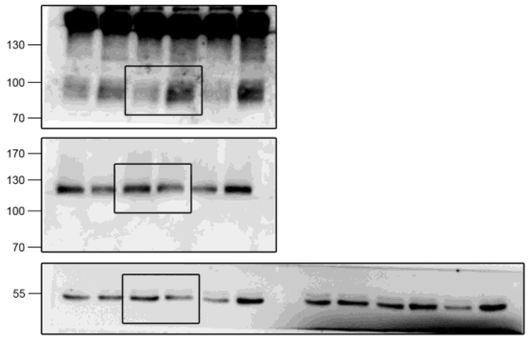

(related to Figure 10)

10A

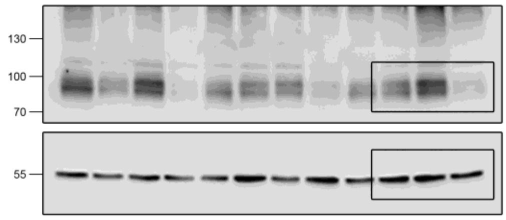

10B

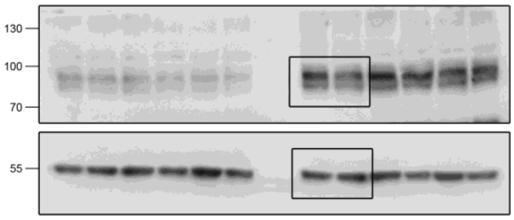

10C

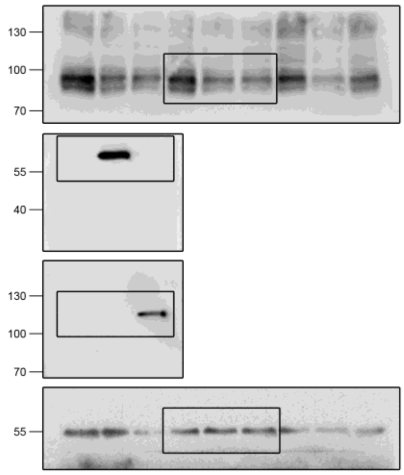

10D

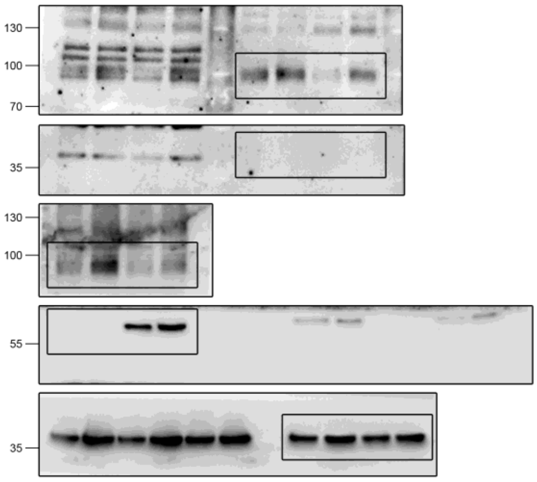

(related to Figure 11)

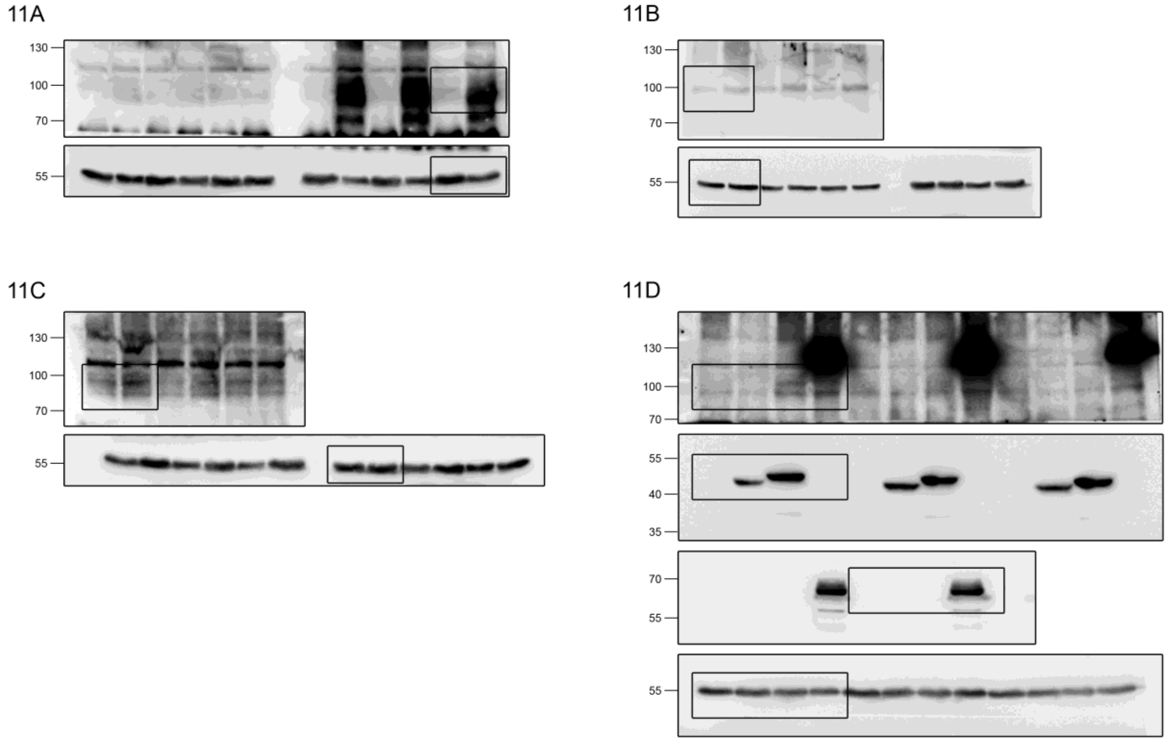

Supplement: Supplementary file 1 [file cells-09-01332-s001.pdf]
